# Development and application of a next-generation sequencing protocol and bioinformatics pipeline for the comprehensive analysis of the canine immunoglobulin repertoire

**DOI:** 10.1371/journal.pone.0270710

**Published:** 2022-07-08

**Authors:** Jonah N. Cullen, Jolyon Martin, Albert J. Vilella, Amy Treeful, David Sargan, Allan Bradley, Steven G. Friedenberg

**Affiliations:** 1 Department of Veterinary Clinical Sciences, University of Minnesota College of Veterinary Medicine, St. Paul, Minnesota, United States of America; 2 Wellcome Trust Genome Campus, Hinxton, Saffron Walden, United Kingdom; 3 PetMedix Ltd, Glenn Berge Building, Babraham Research Campus, Cambridge, United Kingdom; 4 Department of Veterinary Medicine, Madingley Road, Cambridge, United Kingdom; 5 Department of Medicine, Jeffrey Cheah Biomedical Centre, Cambridge, United Kingdom; National Research Council Canada, CANADA

## Abstract

Profiling the adaptive immune repertoire using next generation sequencing (NGS) has become common in human medicine, showing promise in characterizing clonal expansion of B cell clones through analysis of B cell receptors (BCRs) in patients with lymphoid malignancies. In contrast, most work evaluating BCR repertoires in dogs has employed traditional PCR-based approaches analyzing the IGH locus only. The objectives of this study were to: (1) describe a novel NGS protocol to evaluate canine BCRs; (2) develop a bioinformatics pipeline for processing canine BCR sequencing data; and (3) apply these methods to derive insights into BCR repertoires of healthy dogs and dogs undergoing treatment for B-cell lymphoma. RNA from peripheral blood mononuclear cells of healthy dogs (n = 25) and dogs newly diagnosed with intermediate-to-large B-cell lymphoma (n = 18) with intent to pursue chemotherapy was isolated, converted into cDNA and sequenced by NGS. The BCR repertoires were identified and quantified using a novel analysis pipeline. The IGK repertoires of the healthy dogs were far less diverse compared to IGL which, as with IGH, was highly diverse. Strong biases at key positions within the CDR3 sequence were identified within the healthy dog BCR repertoire. For a subset of the dogs with B-cell lymphoma, clonal expansion of specific IGH sequences pre-treatment and reduction post-treatment was observed. The degree of expansion and reduction correlated with the clinical outcome in this subset. Future studies employing these techniques may improve disease monitoring, provide earlier recognition of disease progression, and ultimately lead to more targeted therapeutics.

## Introduction

B-cell receptors (BCRs), with antibodies as their secreted form, are an essential part of adaptive immunity. They are formed by the somatic recombination of variable (V), diversity (D), and joining (J) genes from the immunoglobulin heavy (IGH) locus and V and J genes from either the immunoglobulin lambda (IGL) or immunoglobulin kappa (IGK) locus. The underlying diversity of the V, D, and J genes, junctional diversification, VH/VL pairing and further processes such as somatic hypermutation (SHM) and gene conversion, combine to generate a highly diverse repertoire of B cells, each with a distinct somatically-encoded BCR [1, 2]. The potential diversity of >10^13^ unique BCRs confers the ability to recognize and bind an immense number of foreign antigens [3, 4].

The earliest work to evaluate BCR repertoires began with Southern blots, progressed through a PCR-based approach, and now employs NGS as the most specific method for capturing the breadth of the expressed repertoire [5, 6]. The first iterations of BCR NGS methods included both wholly unbiased bulk sequencing and targeted sequencing using specific primer pairs [7, 8]. In the former case, immunoglobulin reads can make up as little as 1% of the total sequenced dataset leading to comparatively low coverage V(D)J segments and a reduced likelihood of capturing rare clonotypes [7]. The targeted approach leads to greater specificity, but given the variation that exists at the 5’ end of an antibody sequence due to both germline diversity and somatic hypermutation, it is challenging to evenly sample the full repertoire without introducing technical bias or the use of very large pools of forward primers, which can be challenging in species whose immunoglobulin repertoire annotation is incomplete. In contrast to these earlier methods, 5’ rapid amplification of cDNA ends (5’ RACE) serves as a more balanced method of sampling the antibody repertoire [9]. Through the use of a primer or primers directed at the constant region of the antibody chains and a reverse transcription step that leaves known sequence at the 3’ end of the cDNA, it is possible to capture the full V(D)J sequence without the need for gene-specific forward primers. 5’ RACE has largely become the standard method for sequencing BCR repertoires, and has been widely applied in human and murine immune repertoire analysis [10, 11]. Additionally, the introduction of unique molecular identifiers (UMIs) [12] to track RNA molecules through sequencing and analysis has led to significant advances in the quantification and profiling of immune repertories [13–17].

In canine immunology, most work evaluating BCR repertoires has employed PCR-based targeted approaches to analyze the IGH locus in both healthy dogs and dogs with lymphoproliferative disorders [1, 18–23]. More recently, some researchers have started to apply next generation approaches to analyze canine BCR repertoires. The first approach used a PCR-based strategy to amplify 1200 heavy and 500 light chain transcripts from dogs of various sizes, before using NGS to sequence them for analysis of their repertoires [1]. Another study combined a 5’ RACE protocol with traditional Sanger sequencing to characterize the IGH repertoire in three healthy dogs [24]. A more recent study used a targeted PCR approach coupled with NGS to evaluate the IGH, TRB, and TRG loci in a single dog with T cell-rich large B cell lymphoma [25]. However, to the best of our knowledge, there have been no published studies to date examining the entire BCR repertoire including IGH, IGK, and IGL chains using an unbiased high-throughput sequencing approach in populations of dogs.

In this study, we sought to apply state-of-the-art methods, developed to evaluate human and mouse BCR repertoires, to the analysis of the canine BCR repertoire. Our objectives were as follows: (1) to develop a reliable 5’ RACE protocol for generating NGS libraries for evaluating populations of canine B cells, (2) to develop an open-source bioinformatics pipeline for processing canine antigen receptor sequencing data, and (3) to apply these sequencing and bioinformatics methods to derive insights into the BCR repertoires of healthy dogs and dogs undergoing treatment for intermediate-to-large B-cell lymphoma, a common canine hematopoietic neoplasm that we used as a model for B-cell clonality.

## Materials and methods

### Study population and sample collection

We recruited apparently healthy dogs of any breed presenting to the University of Minnesota Veterinary Medical Center (UMN-VMC) Primary Care Service for a routine wellness appointment. All dogs were required to have had a physical examination performed by a veterinarian prior to enrollment. Exclusion criteria were as follows: history of vaccination within the past 30 days, currently taking any medication that might alter immune system function (e.g., prednisone, cyclosporine), moderate to severe anemia (hematocrit <20%), severe thrombocytopenia (platelet count <20 x 10^9/uL), or weight <10 kg.

Concurrently, we recruited dogs with a new diagnosis of intermediate-to-large B-cell lymphoma presenting to the UMN-VMC Medical Oncology Service with an intent to pursue single- or multi-agent chemotherapy. Diagnosis of B-cell lymphoma was made based upon a peripheral lymph node aspirate or biopsy interpreted by a board-certified veterinary pathologist as well as flow cytometry or PCR for antigen receptor rearrangement (PARR) documenting a clonal population of B cells using lymph node aspirates. Exclusion criteria were as follows: prior treatment with any chemotherapeutic agent (including prednisone), primary extranodal lymphoma without lymph node involvement (e.g., hepatic, mediastinal, gastrointestinal lymphoma), moderate to severe anemia (hematocrit <20%), severe thrombocytopenia (platelet count <20 x 10^9/uL), or weight <10 kg.

For healthy dogs, 8 mL of peripheral blood was drawn at a single time point into a sodium citrate CPT tube (BD Biosciences) and peripheral blood mononuclear cells (PBMCs) were immediately isolated following the manufacturer’s protocols. For dogs with B-cell lymphoma, 8 mL of peripheral blood was drawn into an identical CPT tube immediately prior to commencing chemotherapy, and again approximately six weeks later; PBMCs were similarly isolated at each time point. From all dogs, RNA was immediately isolated using the NucleoSpin RNA Kit (Macherey Nagel) and frozen at -80°C. RNA samples were batch shipped overnight on dry ice for library preparation and sequencing.

This study received prior approval from the Institutional Animal Care and Use Committee at the University of Minnesota (Protocol ID: 1612-34378A, approval date: December 20^th^, 2016). Owners of all animals consented to their inclusion in this study.

### 5’ RACE protocol

**Fig 1:**
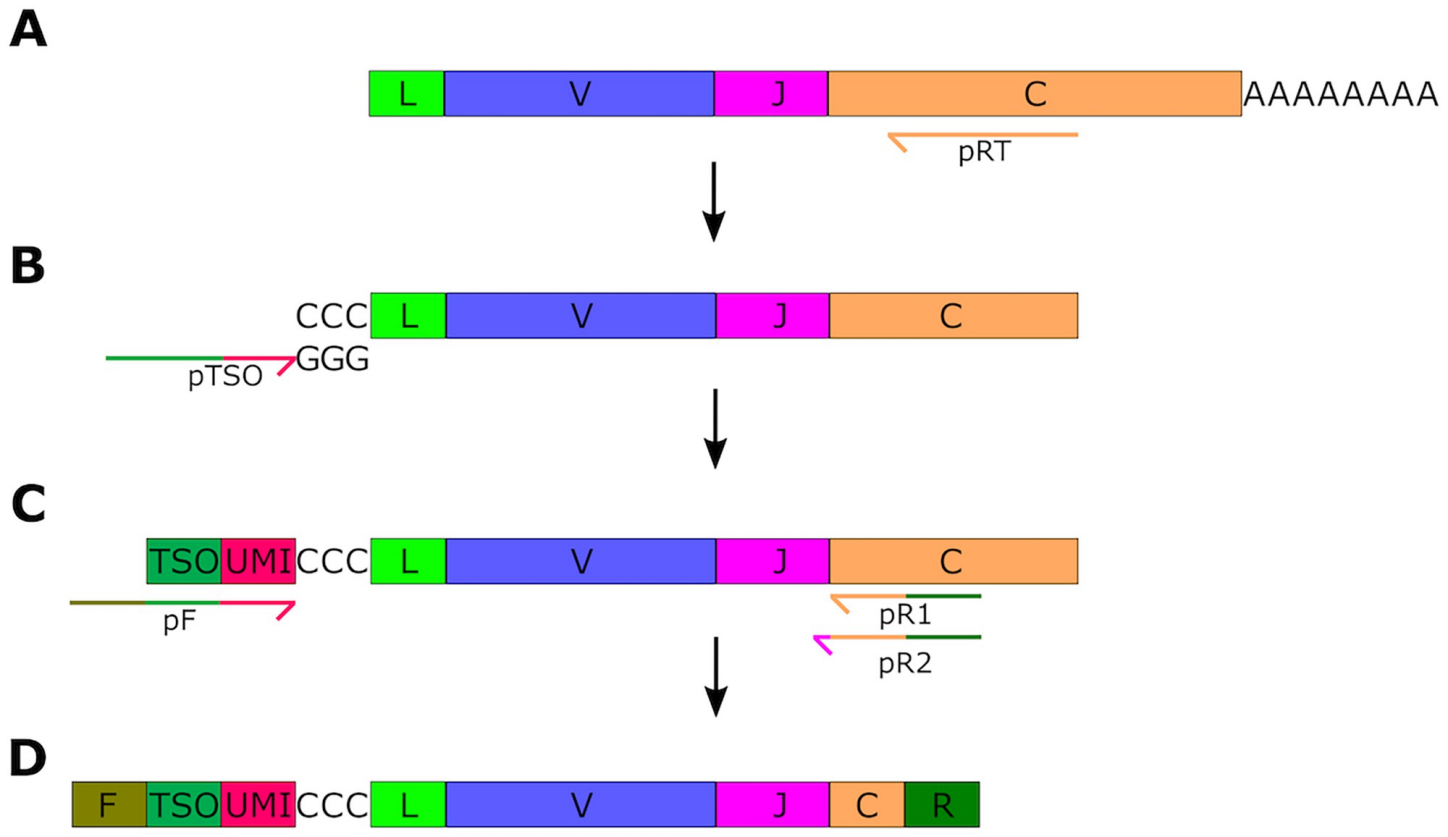
Library preparation using 5’ Rapid Amplification of cDNA Ends (5’ RACE). (A) Antigen receptor mRNA is reverse-transcribed using primers (pRT) that anneal to the constant region (orange box), using a polymerase that adds non-templated cytidines to the 3’ end of the cDNA. (B) The template switch oligo (pTSO) then anneals to these cytidines and provides a template that is extended by the reverse-transcriptase to yield a reverse strand cDNA copy of the transcript incorporating the TSO sequence. (C) This template is then amplified using a forward primer (pF) that anneals to the TSO sequence (green box), upstream of the UMI (red box) added by the TSO, and reverse primers (pR1 and pR2) that anneal to the constant region sequence adjacent to the JC boundary. Reverse primers anneal flush to the JC boundary (pR1) or include a few nucleotides of J region sequence (pR2). The primer sequences also include the adapter sequence for the sequencing platform, in this case the Illumina adapters (green region on pF, pR1, and pR2). (D) Amplification yields a product that spans the V(D)J region and a small amount of C sequence, with a UMI at the 5’ end and adapter sequences at both ends (F and R, green boxes).

We developed a 5’ RACE protocol to create NGS libraries from RNA to capture each BCR repertoire. This process involves four steps (Fig 1): simultaneous reverse transcription (Fig 1A) and template switch (Fig 1B) steps, which converts RNA to cDNA and adds a poly-C header and unique molecular identifier (UMI) to the 5’ end of the sequence (NB: this is the 3’ end of the cDNA); followed by two rounds of PCR amplification (Fig 1C) that add sequencing adapters to each end. These steps yield final libraries ready for sequencing (Fig 1D). Following each step, the reaction mixture was cleaned to remove buffers, enzymes, and other reagents, ensuring that only the nucleic acid product moves downstream.

The template switch step makes use of a template switch oligonucleotide (TSO) that contains 8 random bases; these random nucleotides serve as a unique molecular identifier (UMI). Because UMIs are incorporated at the first step in library preparation, they represent the result of a single reverse transcription reaction. Assuming that each RNA molecule is transcribed once during library preparation, the UMI serves as a tag for a unique RNA molecule. UMIs were used in downstream processing to assess how many sequencing reads were linked to each original cDNA strand, providing a measure of sequencing depth. Additionally, if multiple sequencing reads per were UMI identified, they were collapsed to call a consensus read, which allows for correction of PCR and sequencing errors (see Sample Processing below).

For the concurrent reverse transcription and template switch steps, first strand cDNA was synthesized with a mixed pool of constant region-specific primers, one to each of the canine constant regions (S1 Table: cIGHA-RT to cIGLC-RT), and a template switch oligo (S1 Table: TSO). For the constant region-specific primers, the four heavy chain primers (S1 Table: cIGHA-RT to cIGHM-RT) were pooled in an equal molar ratio to create a heavy chain RT primer mix. Similarly, the two light chain primers (S1 Table: cIGKC-RT and cIGLC-RT) were pooled in an equal molar ratio to create a light chain RT primer mix.

The sequencing reaction contained: 1–2 μg RNA, 667 μM dNTPs (Sigma Aldrich), 667 nM TSO, 333 nM of each of the heavy and light chain RT primer mixes, 2 mM DTT (Invitrogen), 3 mM MgCl_2_, 40 units of RNaseOUT (Invitrogen), and 100 units of SuperScript II reverse transcriptase (Invitrogen). Extension was performed at 42°C for 60 minutes, after which 1 unit of RNase A/T1 mix (Thermo Fisher) was added and the reaction was incubated at 37°C for 15 minutes. The reaction was then cleaned up using the AMPure XP system (Agencourt) according to the manufacturer’s instructions using a reaction:AMPure XP solution ratio of 5:4.

For the first PCR reaction, cDNA from step 2 was resuspended in PCR-grade water and split evenly into two reactions. While the reverse transcription/template switch step is carried out with mixed heavy and light chain primers, the next step creates one heavy chain and one light chain library per sample. PCR was carried out using Q5 High-Fidelity DNA Polymerase Master Mix (New England Biolabs), half of the resuspended cDNA, and 400 nM PCR1 forward primer (S1 Table: PCR1-F), and 400 nM of either the heavy chain PCR1 mix (SN Table: cIGHA-PCR1 to cIGHM-PCR1) or light chain PCR1 mix (SN Table: cIGKC-PCR1 and cIGLC-PCR1). Cycling conditions were as follows: 98°C 30 seconds; then 20 cycles of 98°C 10 seconds, 63°C 30 seconds, 72°C 20 seconds; followed by a final extension at 72°C for 2 minutes. The PCR reactions were subjected to the same AMPure XP cleanup as described above and resuspended in PCR-grade water.

The second PCR reaction was prepared by creating a 50/50 mixture of the first PCR reaction and Q5 polymerase Master Mix, along with 400 nM each of the PCR2 reverse primer (S1 Table: PCR2-R) and a forward primer containing a sample-specific index hexamer/barcode (S1 Table: PCR2-I1 to PCR2-I16). PCR2 reverse primers that extend into the J region sequence help promote the amplification of correctly spliced transcripts. The J region nucleotides included in the primers are ones that are fully shared among all the known J regions to ensure that no bias to any individual J gene segment is introduced. The index primer is used to identify the sample, and so the heavy and light chain reactions from one sample should use the same index primer, but an index primer should not be shared between multiple samples. This allows pooling of multiple samples into a single sequencing reaction. Thermal cycler parameters were identical to the first PCR reaction. The product was cleaned up using the AMPure XP system as before and resuspended in PCR-grade water. Libraries were quantified and pooled in an equimolar mix and diluted to a final concentration of 10 nM for sequencing.

### Sequencing

Heavy and light chain libraries from an average of 8 dogs (range: 6–10) were pooled. Each pooled reaction was sequenced on a single flow cell of an Illumina MiSeq sequencer in a 300 base-pair paired-end read configuration with a including 10% PhiX spike-in by the core sequencing facility at the Wellcome Trust Sanger Institute. The sequencing strategy resulted in between one and six sequencing runs per dog per time point. De-multiplexed FASTQs were concatenated by read orientation per sample and time point prior to analysis. Raw sequence reads are available through the NCBI Short Read Archive (PRJNA790470).

### Bioinformatics pipeline

We developed an analysis pipeline based upon a previously reported protocol [13] to profile the BCR repertoire using the Snakemake workflow management system [26]. The pipeline utilizes MiGEC [14] for UMI-based error correction of raw sequencing reads (pre-processing) and consensus assembly, MiXCR [16] for germline alignment against the ImMunoGeneTics (IMGT) database [27] and clonotype assembly, and VDJtools [28] for repertoire characterization and analysis (e.g. basic statistics including clonotype counts and frequencies, V(D)J segment usage, spectratyping, Complementarity-Determining Region 3 (CDR3) physical and biochemical property profiles, and diversity statistics). VDJtools employs a resampling strategy for calculating diversity statistics in which clone sets are down-sampled to the size of the smallest clone set among a group of samples, and diversity is calculated; this process is repeated 1,000 times resulting in both a mean and standard deviation for diversity statistics. VDJtools also removes clonotype sequences with frameshift mutations and stop codons (non-functional clonotypes) which are saved separately. The pipeline also parses standard MiGEC/MiXCR pre-processing, alignment, and assembly logs into tables for further analyses and quality control. Additionally, it performs mapping and germline assignment using IgBLAST followed by the Change-O “MakeDb” method for additional analyses if required, however we did not use any outputs from IgBLAST or Change-O in our work [29, 30]. The analysis pipeline is publicly available (https://github.com/jonahcullen/Wrappar); detailed descriptions of the methods and parameters of individual software packages are available in the documentation for MiGEC (https://migec.readthedocs.io/en/latest/index.html), MiXCR (https://mixcr.readthedocs.io/en/master/index.html), and VDJtools (https://vdjtools-doc.readthedocs.io/en/master/index.html).

### Sample processing

FASTQs were pre-processed for each dog, time point, and isotype using UMI-based error correction and assembly into error-corrected sequences via MiGEC. Error correction is achieved by aggregating sequences with identical UMIs together into molecular identifier groups (MIG). Due to the range in sequencing depth across sample time points, a threshold of three reads per UMI (e.g. MIG size of three) was chosen to allow for the analysis of larger BCR repertoires at the expense of more accurate error correction (e.g. MIG size > 10). Assembled forward and reverse reads were merged at a minimum 70% similarity (https://github.com/milaboratory/mitools). IGM, IGG, IGA, and IGE assembled reads were concatenated into a single IGH set per sample time point; IGK and IGL reads were maintained as separate datasets.

MiXCR was used to align IGH, IGK, and IGL sequences from MiGEC against the “VRegion’’ reference feature (beginning of FR1 to end of germline V gene) of the IMGT database released in September 2020 (2020038–1) [27]. The top hit per input BCR sequence was retained and assembled using the default CDR3 region. Aligned reads without a CDR3 were dropped. The MiXCR CDR3 feature includes the V region cysteine and J region phenylalanine/tryptophan in its definition of the CDR3. While MiXCR was readily able to identify the CDR3 boundaries of the IGH and IGK genes, it consistently included an extra amino acid from Framework Region 3 (FR3) into the IGL CDR3 (making the cysteine the second amino acid of the CDR3, not the first). To correct this error, IGL CDR3s were manually trimmed prior to analysis. Assembled clonotypes were exported to tables for further analysis.

Clonotype tables were converted into VDJtools format and collapsed by CDR3 amino acid sequence and V/J gene segments for each sample, time point, and chain (IGH, IGK, IGL). Within this work, a clonotype is defined based on three factors: the identified V gene, the identified J gene, and the CDR3 amino acid sequence. While there is not one single standardized definition of a clonotype, our definition is consistent with others in the literature, and was used across all clonotype analyses [31]. For quality assurance, sample-time point-chain combinations with less than 1,000 error-corrected reads or where greater than 95% of reads failed to define clonotypes were removed from downstream analyses. Diversity statistics were calculated for all sample-time-chain combinations that met the inclusion criteria.

### Healthy dog BCR repertoire

#### Gene segment usage

The proportions of V and J gene segment usage among functional clones for IGH, IGK, and IGL were evaluated using the default output from VDJtools’ “CalcSegmentUsage” method. This function applies Euclidean distance-based hierarchical clustering of V and J gene usage profiles to generate tables and heatmaps of gene usage. Weighted V and J gene junction combinations were calculated and circos plots generated using VDJtools’ “PlotFancyVJUsage” method for each individual. Similarly, unweighted V and J gene junction combinations were calculated and alluvial and circos plots generated across combined controls. Unweighted VJ combinations were filtered to include only those greater than 1% and visualized with the R package *ggalluvial* [32]. The R package *Immunarch* was used separately to obtain V(D)J gene counts from clonotype tables in order to summarize by family (e.g. IGHV1, IGHV3, and IGHV4) and calculate normalized proportions (i.e. the per sample gene proportion sum equals 1) for each V, D, and J gene [33]. Spearman’s rank correlation was used between pairwise combinations of IGHV gene usage proportions where gene segments were present in greater than 5% of functional clones.

#### CDR3 length

CDR3 lengths were evaluated using the “ShortCDR3” feature from MiXCR. This feature uses the same definition as IMGT and runs from position 105 to 117 without including the conserved cysteine of FR3 nor the first tryptophan/phenylalanine of FR4 [34]. The distribution of functional clone size was calculated per chain as the average proportion of functional clones for each observed CDR3 length. CDR3 lengths with average proportions less than 0.00001 were excluded to remove likely aberrant CDR3s.

#### CDR3 composition

Common CDR3 lengths were identified for each chain (13, 9, and 11 amino acids for IGH, IGK, and IGL respectively) and the properties of all qualifying CDR3s were considered on a per chain basis. Residues that flank and define the CDR3 were also included, and the positions are referred to by their IMGT position. Prior to calculating properties per position, CDR3s were filtered by length to include only CDR3 and flanking residues (i.e., 15 for IGH, 11 for IGK, and 13 for IGL). Sequence logos were generated for each chain using these lengths with the R package *ggseqlogo* [35, 36]. The amount of information contributed (bits) using Shannon entropy is represented by amino acid letter height as implemented in *ggseqlogo* and originally described [37]. Reported properties at each CDR3 position, including hydropathy, size, chemical class, charge, hydrogen donor or acceptor class, and polarity were calculated based upon the proportion of amino acids at each position averaged across included samples using standard biochemical properties [34].

### BCR repertoire in dogs with lymphoma

#### Gene segment usage

As with healthy dog samples, the proportions of V and J gene segment usage among functional clones for IGH, IGK, and IGL were calculated using the default output from VDJtools’ “CalcSegmentUsage” function. We compared gene segment usage between populations in two separate scenarios: (1) cases sampled before chemotherapy (T1 cases) vs. healthy controls and (2) T1 cases vs. cases sampled after chemotherapy (T2 cases).

#### CDR3 length and composition

CDR3 lengths of dogs with lymphoma at T1 and T2 were evaluated in the same manner as healthy samples based on the IMGT positions of 105 to 117, excluding the conserved cysteine of FR3 and first tryptophan/phenylalanine of FR4 [34]. Sequence logos were generated for T1 and T2 time points for IGH, IGK, and IGL as described for healthy control dogs.

#### Repertoire overlap

Clonotype sharing was performed for cases with sequences from the T1 and T2 repertoires passing the quality filtering described above. VDJtools’ “OverlapPair” compares the frequencies of shared clonotypes between samples. VDJtools’ visualization method is limited to the top 100 clonotypes; rather than use this approach, we chose to plot the entire shared repertoire. The Jaccard index (calculated by “OverlapPair” method) was reported as it represents the proportion of shared clones across time points and is commonly employed in repertoire overlap analyses [38–41]. Overlap was defined as an identical CDR3 amino acid sequence with matching V and J genes.

#### Repertoire diversity

Diversity indices were calculated using the resampling strategy as implemented in VDJtools “CalcDiversityStats” function. For a given set of samples, isotype, and time point (e.g. IGH cases before treatment), each clone set was down-sampled to the size of the smallest clone set and various diversity indices (e.g. Chao1, Efron-Thisted, Shannon-Weiner, and inverse Simpson) were calculated. This process was repeated 1,000 times resulting in both a mean and standard deviation for the statistic. Samples with clone size less than 100 were excluded in order to increase the accuracy of the resampling estimates; this resulted in the exclusion of a single control dog (D02485) for IGH from all analyses requiring diversity estimates. Group-level statistical differences were assessed only for the inverse Simpson index using the Wilcoxon signed-rank test for paired cases (T1 vs T2) and the Mann-Whitney-Wilcoxon test for T1 cases vs controls and T2 cases vs controls. Reported p-values were adjusted using a Bonferroni correction.

Additionally, it has been reported that single diversity measures may return inconsistent results depending on the measure chosen compared to Hill-based diversity profiles which enable a more robust characterization of BCR repertoires [42]. Diversity profiles were therefore generated as described by Greiff *et*. *al*. as a range of weights (alpha values) between 0 and 10 with a step size of 0.2 [42]. Hill-based diversity is not defined at alpha = 1 but is equivalent to Shannon entropy which was calculated using the R package *vegan* [43]. Euclidean distance between diversity profiles was calculated [*dist()* from the *stats* R package] and complete-linkage clustering was performed and visualized with the *ComplexHeatmap* R package [44].

#### Principal component analysis

Principal component (PC) analysis was performed in order to better understand changes in the B cell repertoire across samples between T1 and T2. PCs were calculated using *prcomp* in the *stats* R package based on the weighted mean CDR3 amino acid length, proportion of mutations within the V gene region CDR3, and three diversity metrics. The use of these repertoire properties was based on the observation from Galson *et*. *al*. that B cell subsets can be distinguished based on CDR3 lengths, V gene germ-line mutations, and a measure of clonality (1—Simpson index) [45]. Mutation data were extracted from clonotype tables (filtered for non-functional clonotypes) generated by MiXCR (i.e. not converted to VDJtools format). Two of the diversity statistics (inverse Simpson index and 1—the normalized Shannon-Wiener index) were generated via VDJtools resampling procedure described above and are represented as mean estimates. The third metric, the Gini index, was calculated based on the clonotype counts with the R package *ineq* [46]. These three diversity statistics were chosen as they are commonly used and represent various aspects of diversity and have different interpretations [47–52]. Biplots were constructed using the R package *factoextra* [53].

#### Clonality vignettes

Clonality was assessed and visualized for a subset of cases in two ways: 1) circular packing charts, and 2) circos-style VJ usage plots. For the former, circles of sizes proportional to the frequency of each clonotype are arranged in a non-overlapping layout based on the algorithm described by Wang *et al* and implemented in the R package *packcircles* [54, 55]. VJ usage was visualized using a modified version of the “PlotFancyVJUsage” method from VDJtools which generates circos-style plots of V and J gene pairing frequencies.

## Results

### Animals enrolled

**Fig 2:**
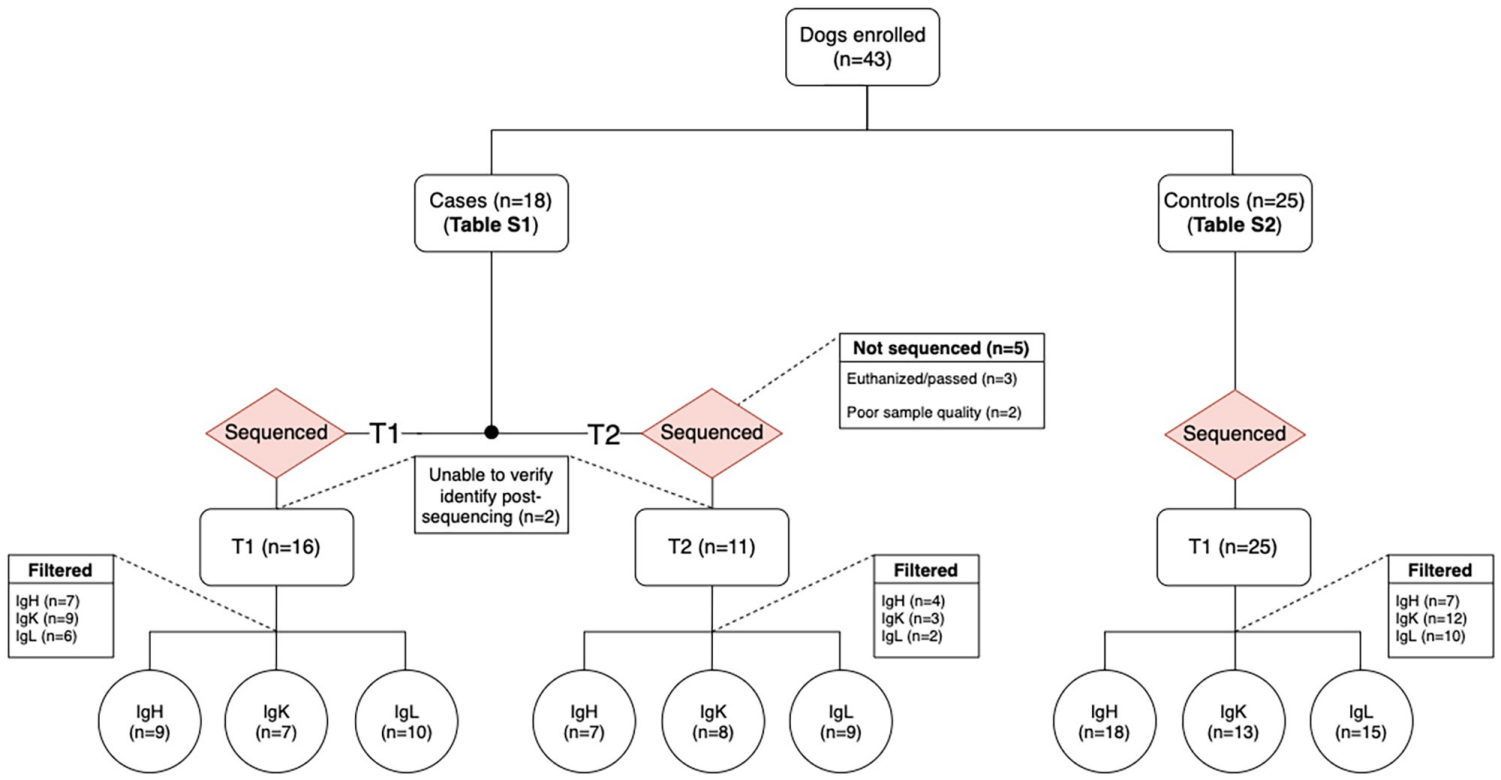
Sample flowchart. Cases and controls enrolled and analyzed across time points and chains (IGH, IGK, and IGL).

Eighteen dogs with intermediate-to-large B-cell lymphoma were enrolled and sequenced (T1 cases). Of the 18 T1 cases, 5 were excluded from post-treatment sequencing (T2 cases) due to poor sample quality (n = 2) or death or euthanasia (n = 3). Two additional dogs were removed from downstream analysis because we could not properly validate their identity after sequencing (Fig 2). For IGH there remained 6 cases with T1 and T2 time points (complete cases), 3 T1 only, and 1 T2 only; for IGK there were 4 complete cases, 3 T1 only, and 4 T2 only; and for IGL 7 complete cases, 3 T1 only, and 2 T2 only for downstream analysis. Twenty-five apparently healthy control dogs were enrolled and sequenced (T1 controls) (Fig 2). Clinical data for cases and controls are available in S2 and S3 Tables, respectively.

### Read and clone filtering

Following concatenation by read orientation, the median (interquartile range) sequence read count was 1,682,922 (907,024^−^2,660,851) across all sequenced cases (n = 18), controls (n = 25), and time points (S1 Fig). Sequencing reads were assembled by UMI and merged via MiGEC and MiTools, respectively, resulting in pools of potential clones (VJ reads) per sample, time point, and chain. The proportions of UMI size per sample-time total VJ reads for each chain is shown in S2 Fig. VJ reads were then aligned and assembled with MiXCR [16]. A threshold was set to exclude chains per sample and time point where the number of VJ reads was less than a 1,000 or greater than 95% of reads were not matched to a clonotype (S3 Fig). This additional filtering step was employed to exclude small and/or low-quality datasets. Excluded chains are listed in S4 Table. Table 1 (cases) and Table 2 (controls) show the total number of functional clones by time point and chain after filtering that were used in downstream analyses. A summary of the UMI-based collapsing and filtering of sequencing reads through total clones (including non-functional) is shown in S4 Fig.

**Table 1:** Signalment and functional clone count of cases.

DogID	Reported Breed	Sex	Age at Enrollment (years)	Clinical Staging		Functional Clones, Enrollment (T1)			Functional Clones, 6 Weeks (T2)		
				Stage	Substage	IGH	IGK	IGL	IGH	IGK	IGL
D02154	Newfoundland	M	5.0	III	a	6,073	189	1,046	8,516	2,029	1,210
D02219	Jack Russell Terrier	F	3.8	III	a	2,578		1,263	951	278	179
D02267^a^	Labrador Retriever	M	11.3	V	b				Euthanized/passed away		
D02269	English Setter	M	10.1	IV	b	2,936	1,398	15,838	2,925	3,108	36,699
D02292	Bassett Hound	F	9.0	III	a		1,247	7,965			
D02386	German Shepherd	F	4.0	III	a						
D02543	Labrador Retriever	F	13.6	III	b		2,430	38,588		2,024	25,577
D02640	Bernese Mountain Dog	M	10.5	IV	b	1,251	1,922	26,262	9,067	2,486	34,324
D02741^c^	Siberian Husky	F	8.7	V	a	3,586			188	692	4,337
D02770^b^	Scottish Terrier	M	9.5	IV	b	7,504	3,756	82,274	Euthanized/passed away		
D02976	American Staffordshire Terrier	F	10.8	V	b	4,169	2,349	17,385	938		1,600
D02977^d^	Rat Terrier	M	9.2	III	a	1,375		2,285		1,019	5,758
D03083	Great Dane	M	4.4	V	b				4,154	2,525	13,259
D03265	Australian Cattle Dog	F	9.5	IV	a				Poor sample quality		
D03631	Weimeraner	F	10.0	III	a	918			Poor sample quality		
D03728^a^	German Shepherd	M	11.6	III	b			210	Euthanized/passed away		

Total number of functional clones by time point and chain after filtering used in downstream analyses for cases. Excluded chains are highlighted in grey.

^a^Euthanized prior 6-week time point (T2).

^b^Passed away prior to 6-week time point from acute tumor lysis syndrome after L-asparaginase therapy.

^c^Intended to pursue cyclophosphamide, doxorubicin hydrochloride (hydroxydaunorubicin), vincristine sulfate (Oncovin), and prednisone (CHOP), but ultimately elected prednisone only.

^d^Intended to pursue CHOP, but ultimately elected prednisone only; lymph nodes were not measured at T2.

**Table 2:** Signalment and functional clone count of controls.

DogID	Reported Breed	Sex	Age at Enrollment (years)	Functional Clones		
				IGH	IGK	IGL
D02293	Labrador Retriever	M	2.8		1,504	16,446
D02318	Labrador Retriever	F	5.6		1,672	23,945
D02348	Labrador Retriever	M	9.3	896		394
D02353	German Shepherd	M	3.6		1,775	15,130
D02354	Labrador Retriever	M	12.3	909		232
D02365	Newfoundland	M	5.3	292	2,086	23,952
D02366	Labrador Retriever	M	11.5	1,100	2,337	39,039
D02447	Golden Retriever	F	12.4	10,774	2,803	43,649
D02482	Labrador Retriever	F	1.6	660		
D02483	Labrador Retriever	M	7	459	169	
D02484	German Shepherd	F	5.3			
D02485	Drahthar	M	6.9	99		
D02486	Labrador Retriever	M	8	3,222		570
D02487	Border Collie	M	4.7	388		
D02488	Cavalier King Charles	M	6.3	1,486		
D02489	Standard Poodle	F	8.4	10,691		
D02493	Golden Retriever	F	9.4	5,621	2,572	20,014
D02942	German Shepherd	F	4.3	5,423	2,870	26,432
D03078	English Setter	M	10		1,384	11,444
D03213	Australian Shepherd	M	8.2	423		
D03291	Catahoula Hound	M	12.4	25,879	4,614	29,238
D03345	Mixed	F	4.3	291	1,300	3,786
D03520	Mixed	M	6.9	5,552	1,105	7,535
D04081	Shih Tzu	M	10.3			
D04390	German Shepherd	M	11.3			

Total number of functional clones by time point and chain after filtering used in downstream analyses for controls. Excluded chains are highlighted in grey.

### Healthy dog BCR repertoire

#### Gene segment usage

**Fig 3:**
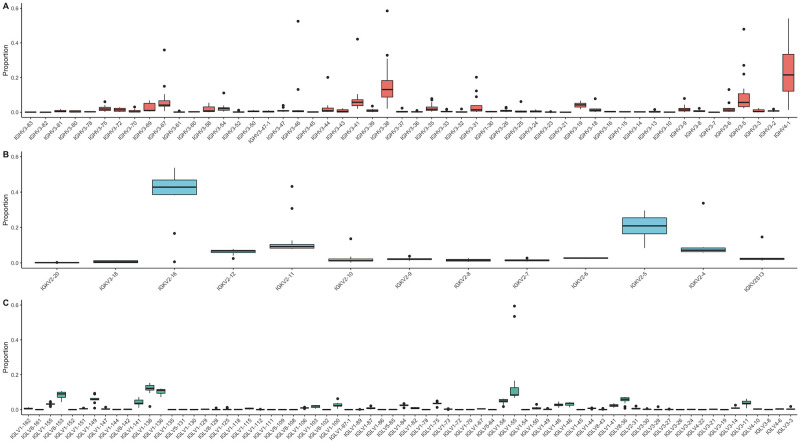
V gene usage. (A) IGHV. (B) IGKV. (C) IGLV. Genes are in locus order from left to right where the constant region(s) would be on the right hand end of the figure (i.e. IGHV3-83 is the most C-distal and IGHV4-1 the most C-proximal gene).

**Fig 4:**
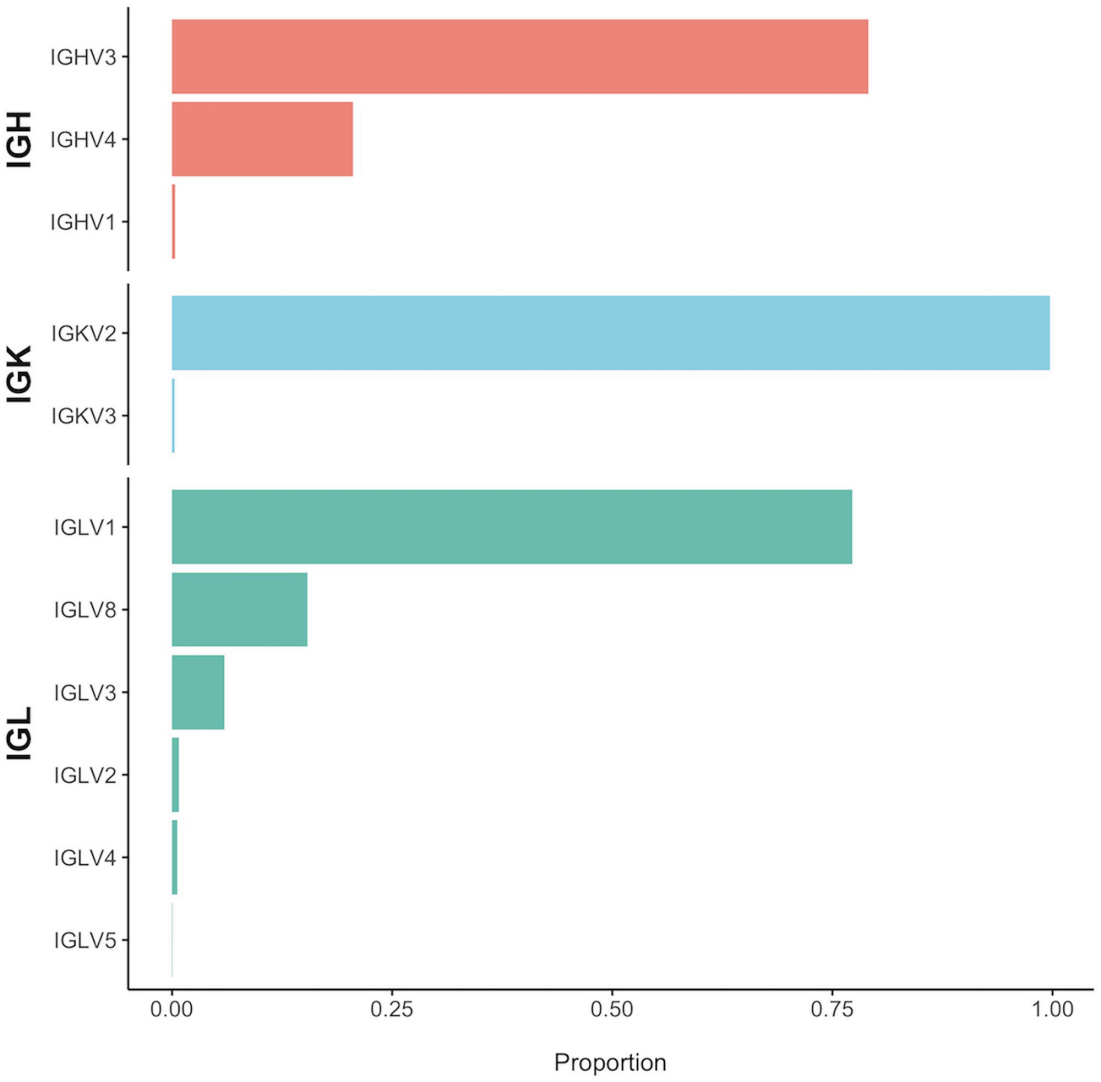
V gene usage by family. Proportion of V gene usage by family for IGH, IGK, and IGL.

*V genes (heavy chain)*. The majority (52/87) of IGHV genes are represented in BCR transcripts of our healthy dog population, with the two most frequently used V genes, IGHV4-1 and IGHV3-38 accounting for 20.6% (15,251/74,165) and 26.1% (19,363/74,165) of V segments in all functional VJ reads, respectively. Other commonly used gene segments present in greater than 5% of rearrangements include IGHV3-41 (11.1%), IGHV3-5 (7.1%), IGHV3-67 (5.6%), and IGHV3-19 (5.5%). As the total proportion may be skewed by samples with greater number of functional VJ reads, Fig 3A depicts the IGHV gene segment proportions per sample. All of the IGHV gene segments not used in our population are pseudogenes with two exceptions: IGHV3-34 and IGHV3-76, both of which are functional. The distribution of IGHV gene segments among functional VJ reads by family is 0.3% (259/74,165) for IGHV1, 79.1% (58,655/74,165) for IGHV3, and 20.6% (15,251/74,165) for IGHV4 (Fig 4). This is consistent with previous findings that identified IGHV3 in 90% of reads, IGHV4 in the rest, and no IGHV1 reads [1]. Among gene segments that are uncommonly used in VJ rearrangements (i.e. present in 5% or fewer rearrangements), no apparent location bias was noted (i.e. there was no preferential usage of C-distal or C-proximal V gene segments, which has been seen in other systems [11]).

**Fig 5:**
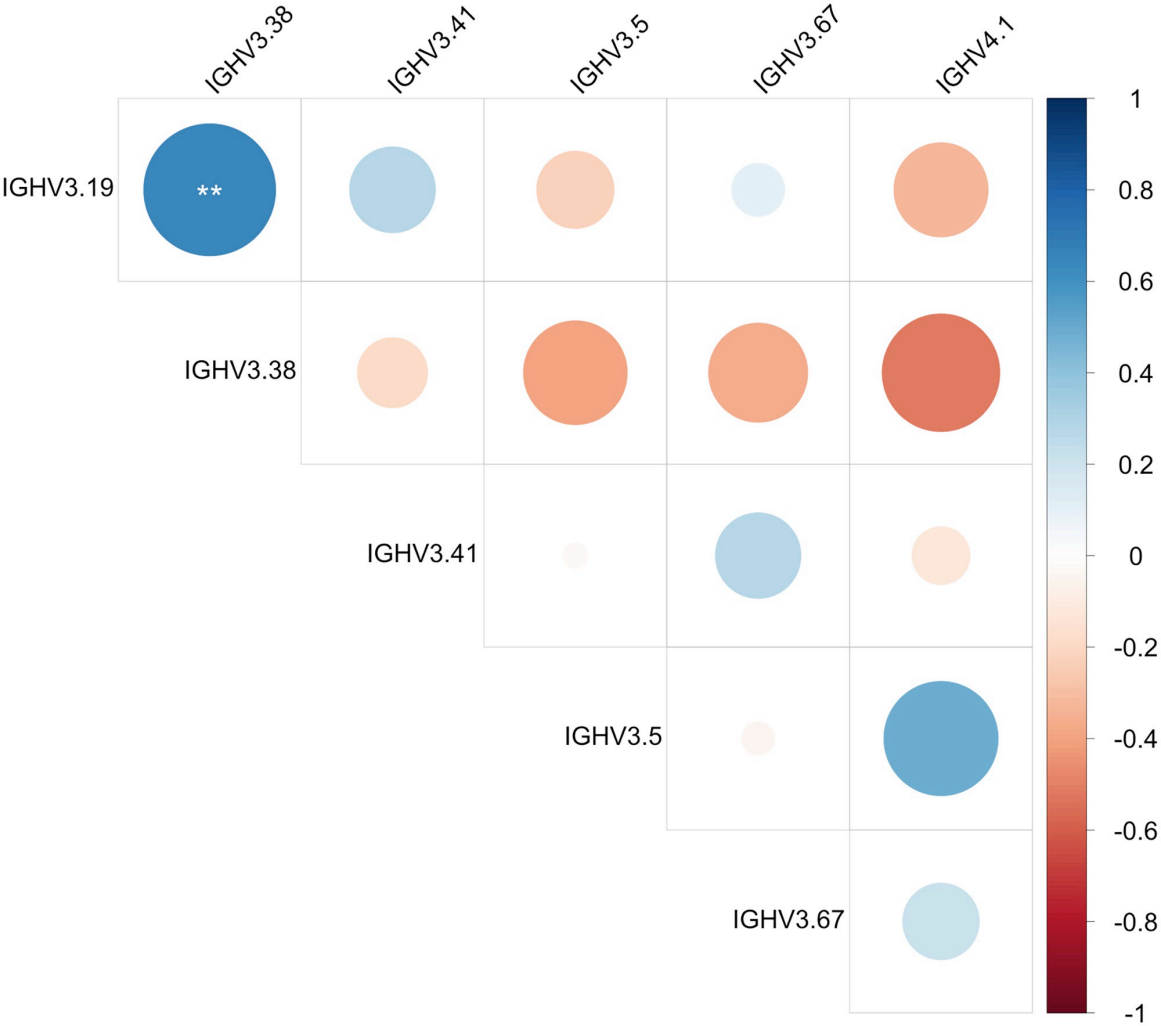
Bi-modal usage of IGHV3-38 and IGHV4-1. Spearman’s rank correlation was used to evaluate pairwise combinations of IGHV gene usage within samples for gene segments present in greater than 5% of functional clones.

Among IGHV gene segments, we observed that individual dogs preferentially used either IGHV4-1 or IGHV3-38 as their most common IGHV at the expense of the other gene. While preferential usage of a specific IGHV gene has been previously observed in certain disease states, a bimodal usage pattern has not been reported before to our knowledge [21]. To evaluate whether there was a significant bimodal usage pattern of these two IGHVs, the observed correlations between IGHV genes present in greater than 5% of rearrangements were calculated and are shown in Fig 5. IGHV3-38 was negatively correlated with IGHV4-1 [Spearman’s ρ = -0.513, 95% CI (-0.737, 0.067)]. IGHV3-19 was significantly correlated with IGH3-38 [ρ = 0.645, 95% CI (0.172, 0.826)].

*V genes (light chains)*. The majority (13/22) of IGKV genes are represented in our healthy dog population (Fig 3B). There is a strong bias toward the use of IGKV gene segments in the IGKV2 family, which together make up 99.7% (26,118/26,191) of V gene segments in functional VJ reads; only 0.3% (73/26,191) of IGKV3 and no IGKV4 family genes are represented (Fig 4). Only five gene segments are present in greater than 5% of rearrangements and include IGKV2-16 (38.7%), IGKV2-5 (20.3%), IGKV2-11 (13.2%), IGKV2-4 (7.6%), and IGKV2-12 (5.7%). Fewer than a third (70/261) of the IGLV genes were found to be expressed (Fig 3C). The canine IGL locus has a large number of pseudogenes, which make up 177 of the 261 genes of the locus; none of these genes was found to be expressed. IGLV1 was the most commonly used family at 77.2% (202,245/261,806), followed by IGLV8 at 15.3% (40,202/261,806), IGLV3 at 5.9% (15,572/261,806), IGLV2 at 0.8% (1,981/261,806), IGLV4 at 0.6% (1,632/261,806), and IGLV5 at 0.07% (174/261,806) (Fig 4). Considering the ratio of IGLV to IGKV expression (261,806/26,191), it has long been reported that dogs are an IGL-biased species, expressing IGLVs 91% of the time [56]. While it is not possible to directly measure the number of canine B cells expressing IGL over IGK in a bulk RNA sequencing experiment, the relative abundance of each mRNA in our work is consistent with this reported ratio (26,191 functional clones with IGKV gene segments vs. 261,806 containing IGLV gene segments).

**Fig 6:**
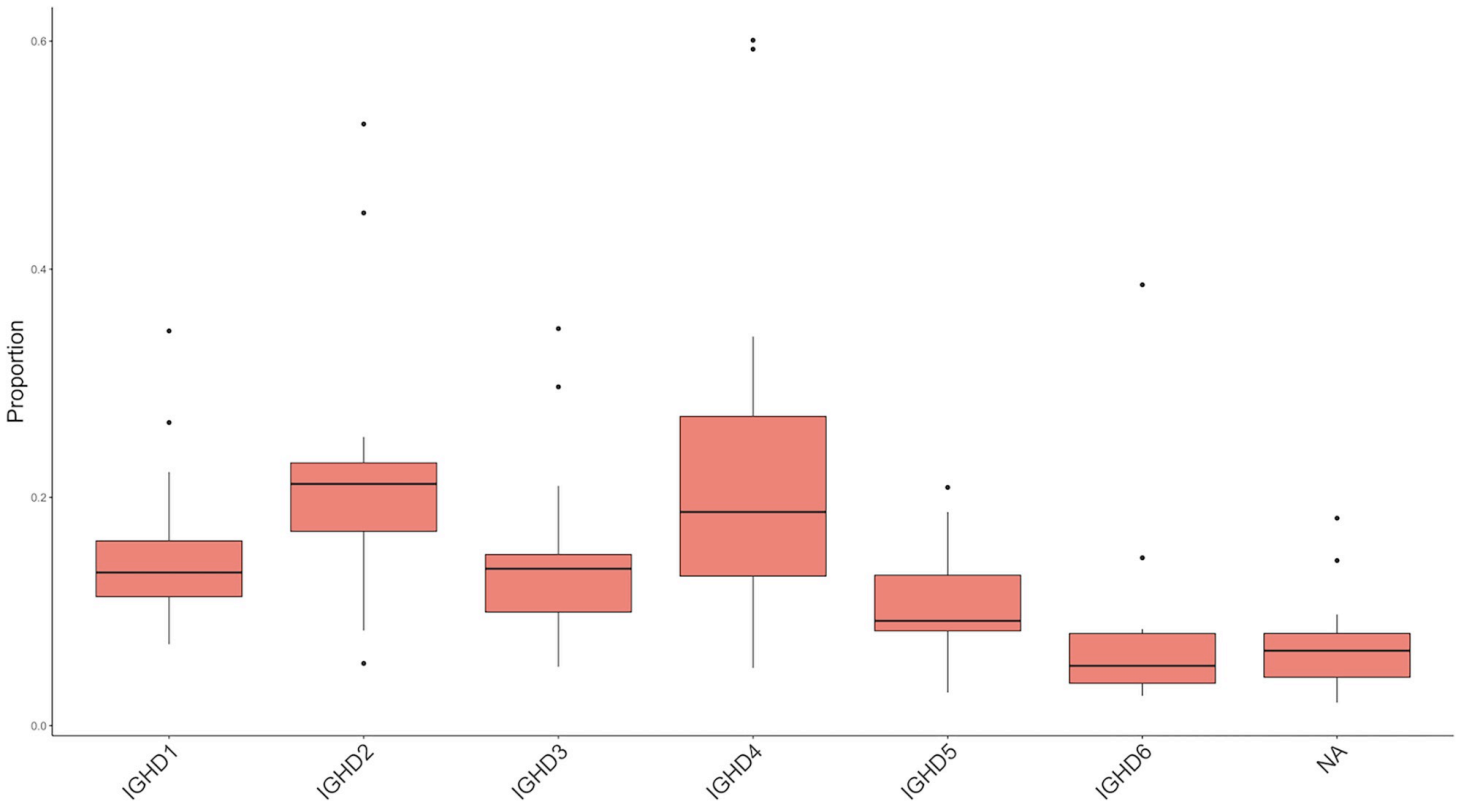
D gene usage. Genes are in locus order from left to right.

*D genes*. All six IGHD genes were found to be used in our control dog population (Fig 6). D genes could not be identified in 9.3% (6,876/74,165) of functional clones, likely due to their small size and ability to be used in multiple reading frames [51, 57]. IGHD4 (24.8%—18,397/74,165) and IGHD2 (22.9%—16,955/74,165) were the two most commonly used IGHD gene segments followed by IGHD1 (13.8% -10,241/74,165), IGHD3 (11.7%—8,660/74,165), and IGHD5 (10.8%—8,043/74,165). IGHD6 was the only gene segment identified in fewer than 10% (6.7%—4,993/74,165) of functional clones.

**Fig 7:**
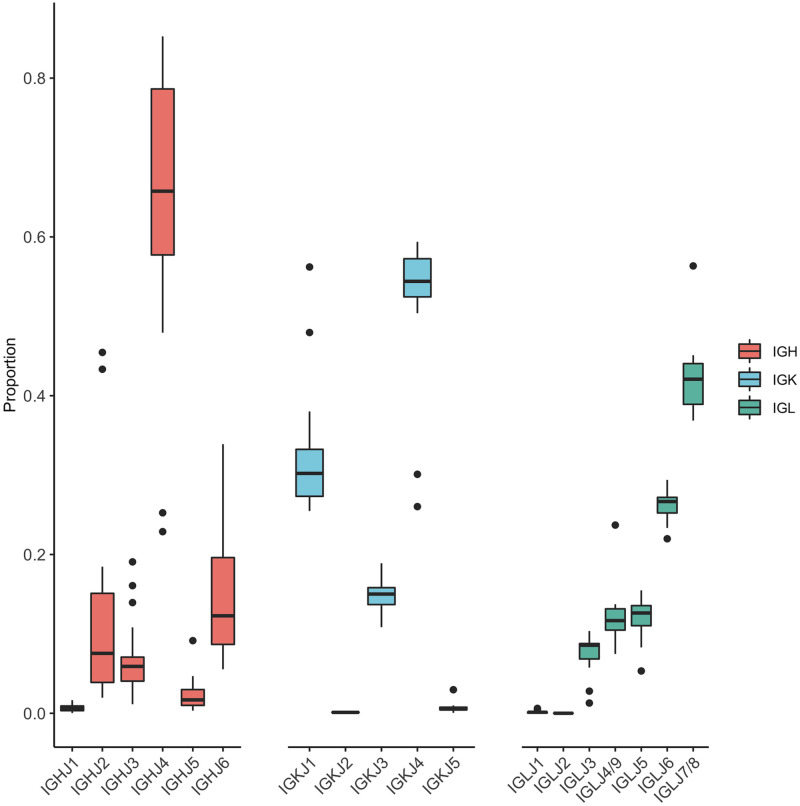
J gene usage by chain. Genes are in locus order from left to right for IGH, IGK, and IGL.

*J genes (heavy and light chains)*. All J genes were found to be expressed, although certain J gene segments were overrepresented (Fig 7). Among heavy chain rearrangements and consistent with previous work, IGHJ4 was the most commonly used IGHJ gene segment (66.6%—49,422/74,165 [J segments/functional VJ reads]) [1, 24]. Among kappa chain rearrangements, IGKJ1 (33.8%—8,853/26,191) and IGKJ4 (50.3%—13,164/26,191) were the most abundant J gene families. Among lambda chain rearrangements, IGLJ7/8 was the most abundant J gene family (40.2% 105,263/261,806). Note that IGLJ4 and IGLJ9, and IGLJ7 and IGLJ8, are identical in their germline sequence. Furthermore, only one IGLC primer was used, which bound very close to the J-C junction in the reverse transcription step so it was not possible to use information from the IGLC segment to identify which IGLJ was used. Therefore, we could not discriminate within each pair and so they were aggregated, meaning it cannot be determined whether usage is even or imbalanced within each pair.

**Fig 8:**
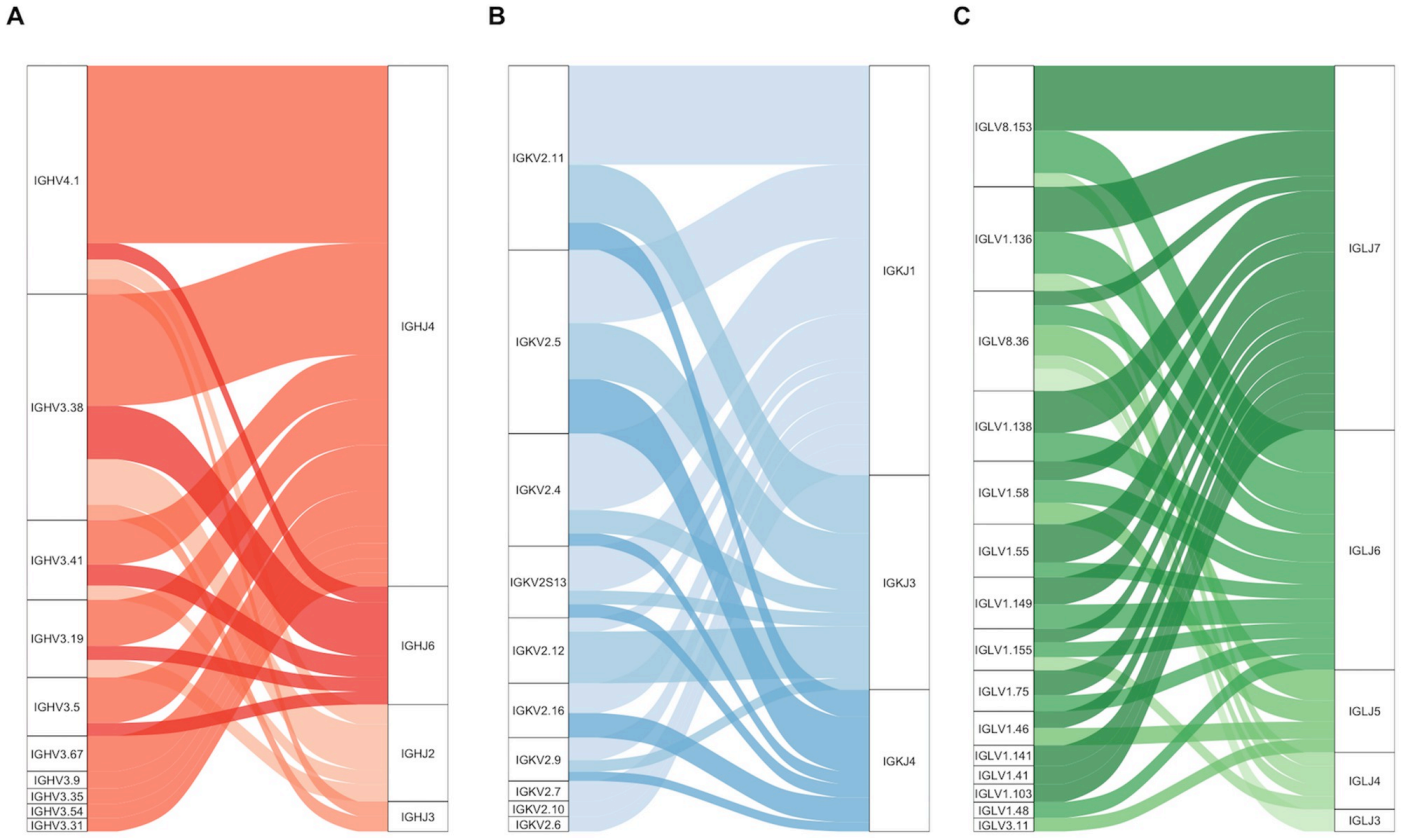
Alluvial plots of paired VJ usage by chain. V and J gene combinations for each of the (A) IGH, (B) IGK, and (C) IGL loci. The thickness of the band joining any pair of V and J genes on the plot represents the unweighted proportion of reads that used that combination of V and J genes across all included controls.

*VJ junction frequency*. Unweighted VJ junction frequencies were calculated across all included controls for IGH, IGK, and IGL. Only V and J gene combinations greater than 1% were included for figure clarity (Fig 8). All possible VJ combinations are shown as Circos plots in S5 Fig. For IGH, IGHJ4 was the most commonly used IGHJ gene and observed most often with IGHV4-1 (15.7%) and IGHV3-38 (9.9%) (Fig 8A). For IGK, IGKJ1 was observed most often with IGKV2-11 (11.9%) and IGKV2-4 (9.2%) (Fig 8B). Among IGL isotypes, there were many more observed VJ junctions, only one of which was observed more than 5% across all included controls, IGLJ7 with IGLV8-153 (5.1%) (Fig 8C).

#### CDR3 length

**Fig 9:**
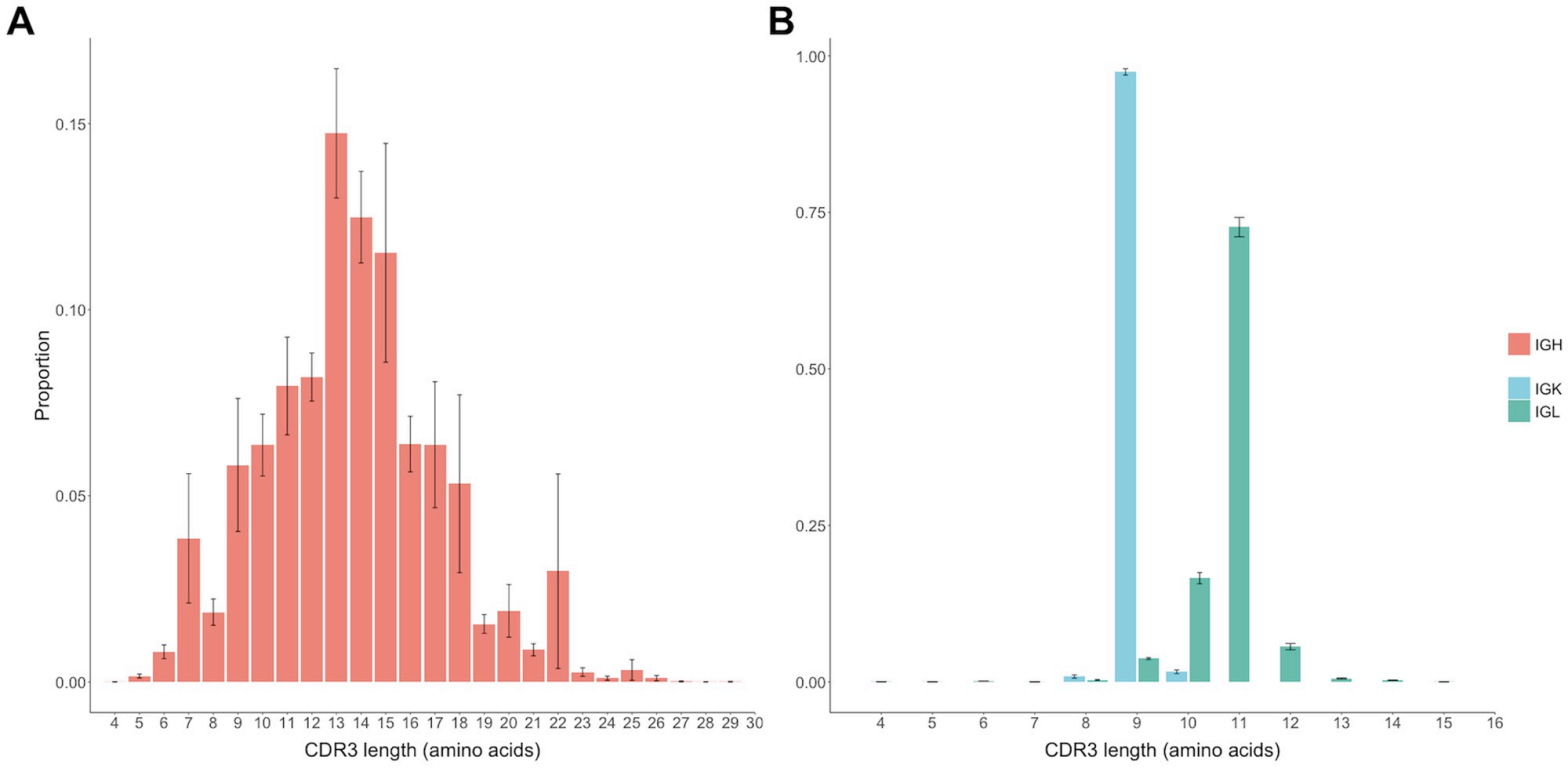
CDR3 lengths for the heavy (A) and light chains (B) of the controls. Distribution of functional clone size was calculated as the average proportion of functional clones for each observed CDR3 length. CDR3 length is defined from position 105 to 117 without the conserved cysteine of FR3 nor the first tryptophan/phenylalanine of FR4.

The distribution of IGH CDR3 (CDR3H) functional reads were most commonly between 13 (14.6%) and 14 (12.4%) amino acids in length, making them similar in length to human CDR3Hs (Fig 9A). Of CDR3Hs, 38.1% were more than 14 amino acids long, mirroring previous observations [1, 24]. They also show the same under-representation of eight amino acid CDR3Hs as mice, and as previously found in dogs [1, 58]. Within the light chains the CDR3 lengths are very restricted; 97.8% of IGK reads are 9 amino acids in length and 70.2% of IGL reads are 11 amino acids in length (Fig 9B). The lack of a D gene reduces potential variability in light chain CDR3 lengths, partly as there is only one junction in which P and N additions can occur, and partly due to variation in the lengths of the D genes themselves.

#### CDR3 composition

CDR loops, and in particular CDR3 loops, are known in humans, mice, and dogs to not have evenly distributed amino acid usage at each position, nor is the variability of amino acid usage (for example, as measured by Shannon entropy) consistent at each position [1, 58]. Our results matched these findings in that they identified uneven amino acid usage, as well as certain positions within the CDR3s that showed strong biases for specific amino acids or groups of amino acids. In order to compare like with like, only CDR3s of the most common length for each chain (IGH = 13, IGK = 9, IGL = 11) were included in our analysis. After filtering CDR3s by length, 10,855 IGH, 25,617 IGK, and 183,902 IGL sequences remained for analysis.

Prior to considering biases at individual positions, the CDR3s were ranked by how frequently they were found across all dogs. Surprisingly, the two most common CDR3K sequences (which differ by a single amino acid) were found to account for 37.2% of all IGK reads (Table 3). This bias tapers off, with less common CDR3Ks appearing in 5% or fewer clones, such that the top three CDR3K sequences account for 42.2% of CDR3Ks. This pronounced bias was not seen for CDR3Ls or CDR3Hs. The three most common CDR3Hs, which make up 7.9% of all CDR3Hs, and are all of different lengths, and the three most common CDR3Ls account for 10.0% of functional reads. Additionally, the most common CDR3K and CDR3L sequences were identified in nearly all included controls; this was not observed for CDR3H sequences, which were typically not shared across dogs.

**Table 3:** Three most common CDR3 sequences by chain.

CDR3 Sequence	Chain	n	Total Count	Fraction
ARTSGAKYWPNNPDY	IGH	1	776	2.90%
AKDSDSSSRYSSSWYPAYNFDY	IGH	2	5,071	2.64%
AREPVHYGTYYAWGNFDY	IGH	1	460	2.32%
QQSLHFPPT	IGK	25	5,873	22.89%
QQSLHLPPT	IGK	13	3,598	14.26%
GQGIQYPFT	IGK	12	1,425	5.03%
STWDDSLSAPV	IGL	27	1,612	4.12%
SSDDDSLSSVV	IGL	16	9,498	3.58%
STWDDSLSAAV	IGL	39	2,107	2.30%

Common CDR3 sequence proportions were weighted across included controls. The number of times the CDR3 sequence was identified across controls is included (n), along with the sum of the CDR3 counts. Note that n can be greater than the number of included controls (18 for IGH, 13 for IGK, and 15 for IGL) as the same CDR3 sequence may identified more than once within a sample with different V and J genes (i.e. a separate clonotype per the described definition above).

**Fig 10:**
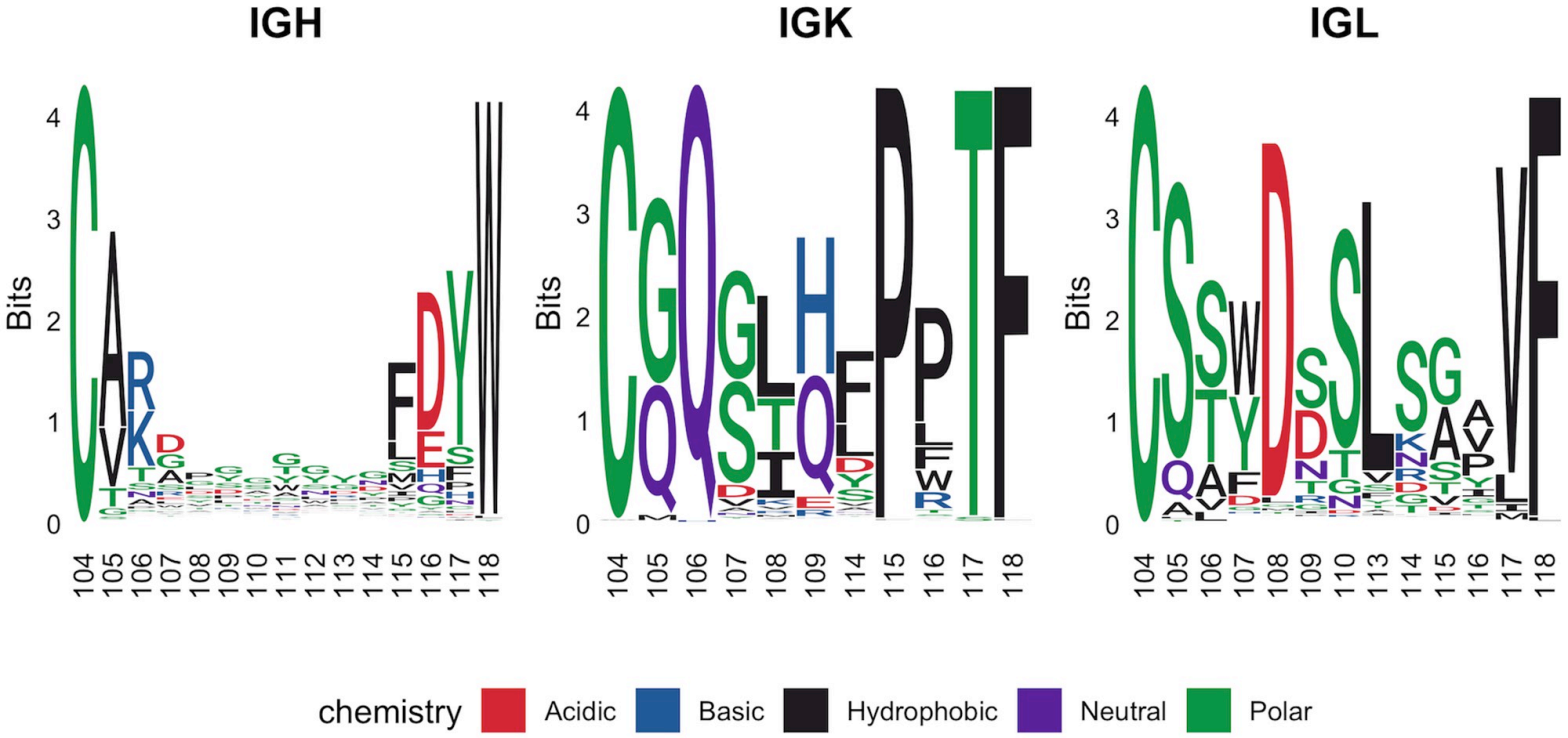
Sequence logos for IGH, IGK, and IGL controls. Only CDR3s of the most common length for each chain (IGH = 13, IGK = 9, IGL = 11) were considered with amino acid letter height scaled to the amount of information contributed (bits) using Shannon entropy [35–37].

Having considered the CDR3s in their entirety, they were then analyzed on a position by position basis (Fig 10). Consistent with other species, the CDR3H positions most skewed to a single amino acid were those at the base of the loop where positions 105, 116 and 117 all showed a greater than 60% usage of a single amino acid. The light chains tell a slightly different story. The biased positions in CDR3L were more evenly distributed over the entire CDR, with positions 108, 113, and 117 all showing greater than 80% preference for specific amino acids. There was also a high prevalence for serine across the entire CDR3L where it was used more than 50% of the time at positions 105, 110, and 114. For CDR3K, there were biased positions as well, but given the overall bias towards a pair of CDR3Ks, individual position biases are dominated by the composition of those two CDR3Ks. The full breakdown of biochemical properties by CDR3 position in our healthy dog population is available in S6 Fig.

### Lymphoma dog BCR repertoire

#### Gene segment usage

V and J gene usage was considered at the group level between (1) controls and T1 cases and (2) T1 cases and T2 controls via Z-score scaling of observed proportions within each sample. Based on the hierarchical clustering, there were no apparent global differences in V gene usage between either group level comparison for IGH (S7 Fig). Notably, paired IGH cases (i.e. cases with T1 and T2 samples) did not necessarily cluster together. As with the healthy controls, IGHV3-38 and IGHV4-1 were the most abundant IGHV genes and IGHJ4 was the most abundant IGHJ gene. For five of the six dogs included here, those that were dominant for IGHV3-38 or IGHV4-1 at T1 were either dominant for the same one at T2 or usage at T2 was balanced. One dog (D02640) was dominant for IGHV4-1 (25.6% of reads) at T1 but was dominant for IGHV3-38 (36.0% of reads) at T2. The absence of V or J gene-based group-level clusters was also observed for IGK and IGL (S7 Fig [V gene segments] and S8 Fig [J gene segments]).

#### CDR3 length and composition

**Fig 11:**
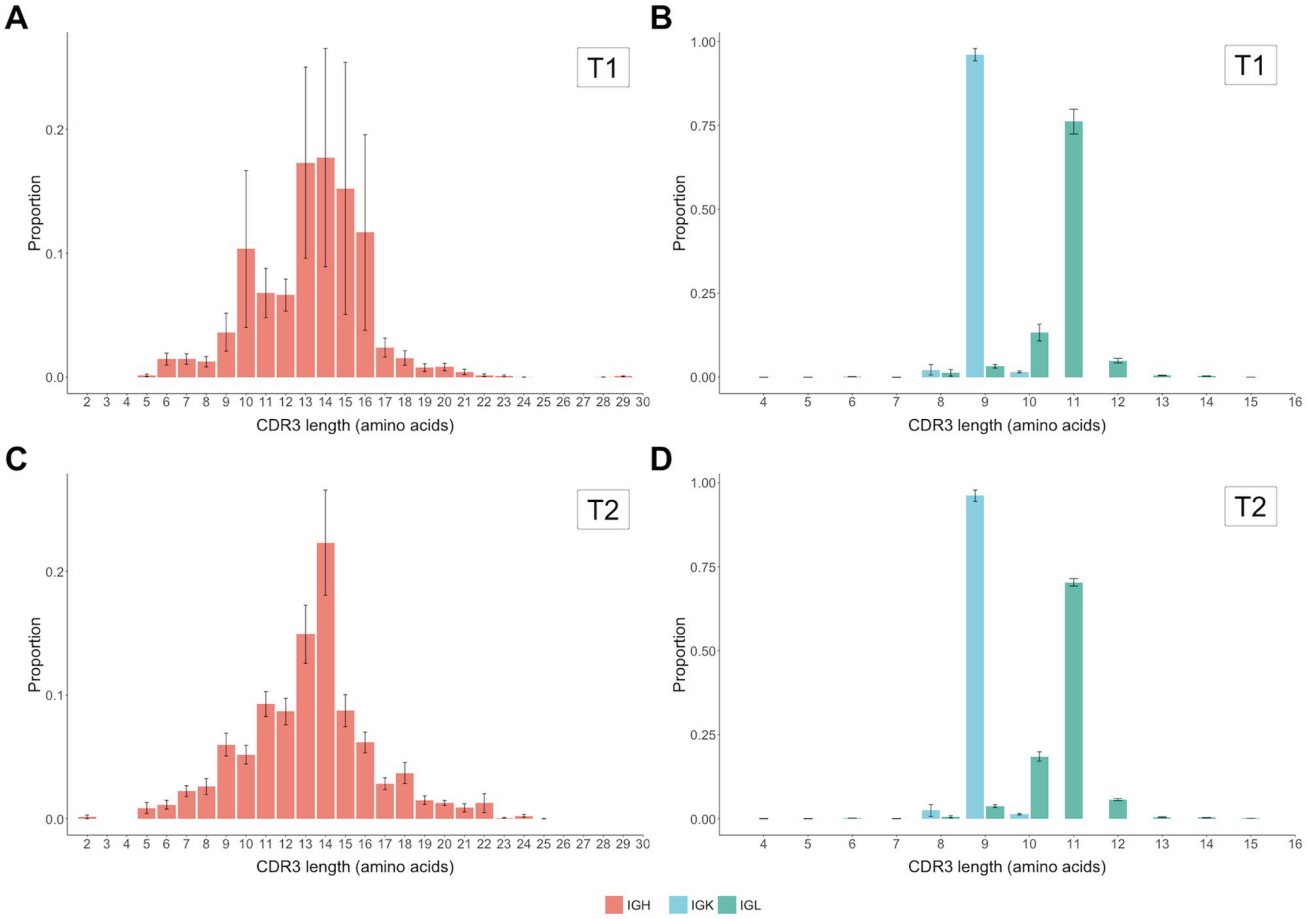
Heavy and light chain CDR3 lengths of cases at T1 and T2. CDR3H amino acid length from case individuals at T1 (A) and T2 (B). Light chain CDR3 lengths from case individuals at T1 (C) and at T2 (D). Distribution of functional clone size was calculated as the average proportion of functional clones for each observed CDR3 length at each time point. CDR3 length is defined from position 105 to 117 without the conserved cysteine of FR3 nor the first tryptophan/phenylalanine of FR4.

**Fig 12:**
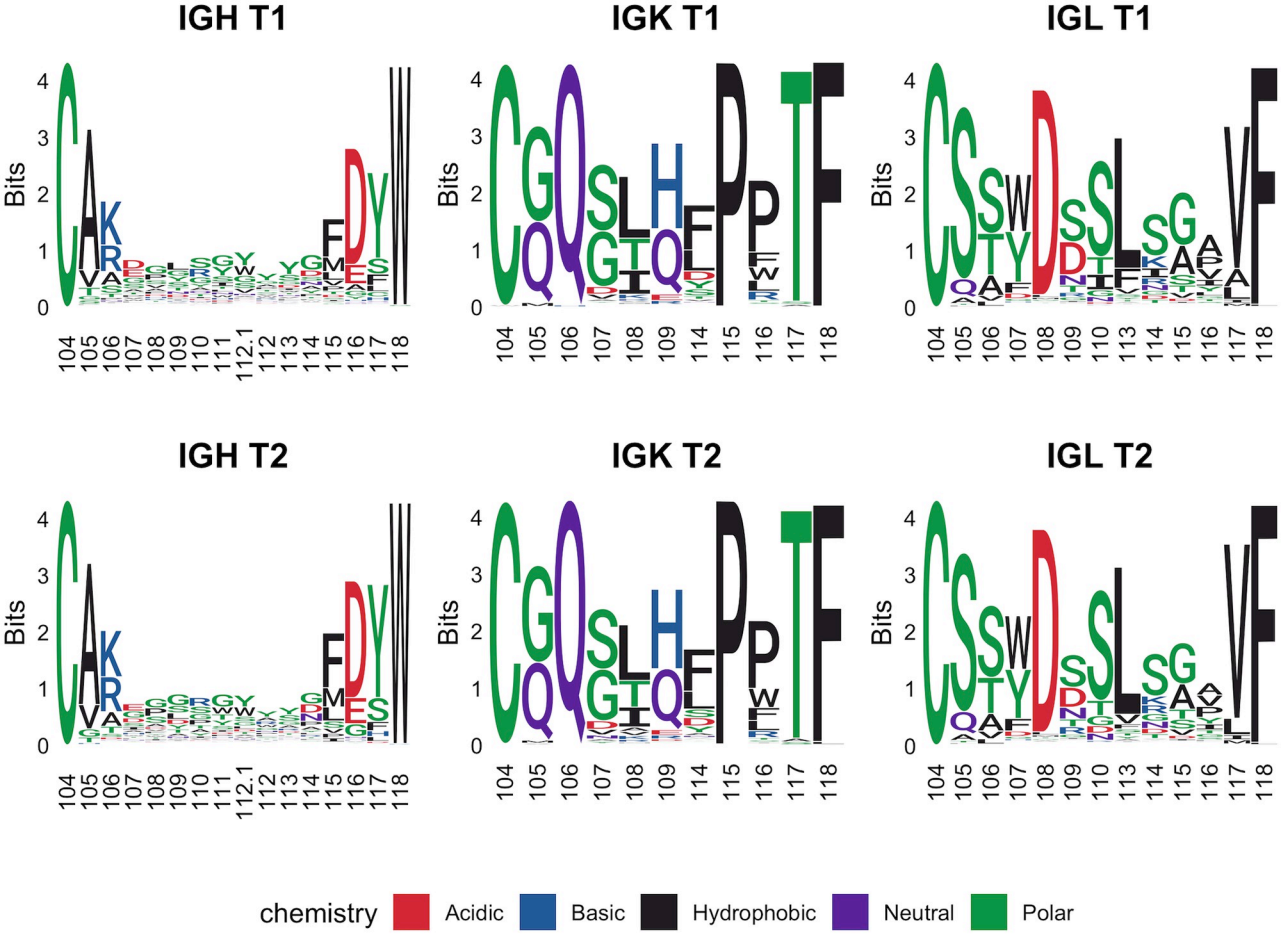
Sequence logos for IGH, IGK, and IGL cases at T1 and T2. Only CDR3s of the most common length for each chain (IGH = 14, IGK = 9, IGL = 11) were considered with amino acid letter height scaled to the amount of information contributed (bits) using Shannon entropy [35–37].

Similar to the healthy controls, the distributions of CDR3H functional reads were most commonly between 13 (T1–17.3%, T2–14.9%) and 14 (T1–17.7%, T2–22.3%) amino acids in length at both time points (Fig 11). Of CDR3Hs, 33.1% of T1 and 26.7% of T2 were more than 14 amino acids long compared to the healthy controls of 38.1%. The under-representation of eight amino acid CDR3Hs noted in the healthy controls was observed to a lesser extent in T1 cases and not at T2. Similar to the healthy controls, the light chain CDR3 lengths are restricted with 96.1% (T1) and 96.2% (T2) of IGK reads at 9 amino acids and 76.1% (T1) and 70.4% (T2) of IGL reads being 11 amino acids in length. There were no notable differences between the T1 and T2 common length CDR3 sequences for IGH, IGK, or IGL (Fig 12). It should be noted that the most common CDR3H length (14) for the case dogs is different from the most common CDR3H length (13) for control dogs and so the CDR3H sequence logos are not directly comparable between cases and controls.

#### Repertoire overlap

Repertoire overlap across time points for three cases (D02154, D02269, and D02640) in which both T1 and T2 time points passed filtering across IGH, IGK, and IGL reads is shown in S9 Fig. Clonotypes with the same CDR3 amino acid sequence and V and J gene segments were considered as overlapping. The regression coefficients were near 1 (1 being perfect overlap of shared clonotypes and frequencies across time points) for IGK and IGL suggesting the overall shared repertoires did not change substantially with treatment. For IGH, where we expected to observe larger differences in shared clonotype frequency, the regression coefficients are well below one for two of the three cases (D02269 and D02640). The larger observed coefficient for D02154 appears to be the result of a single clonotype with a smaller reduction between T1 (84.8%) and T2 (26.4%) relative to the other four shared clonotypes. There was minimal repertoire overlap for IGH between T1 and T2 with proportions of shared clones at 0.4% (D02154), 1.5% (D02269), and 1.3% (D02640) (S5 Table). This was not entirely unexpected for IGH as there were relatively few total clonotypes and thus less potential overlap compared to the light chains. Identified clonotypes were particularly low for D02154 across all chains. Repertoire overlap appeared more consistent for IGK and IGL with shared clones >35% after excluding D02154.

#### Repertoire diversity

For group level comparisons of diversity estimates, we expected no differences between the controls and post-treatment (T2) cases, however we did expect differences in pre- (T1) and post- (T2) treatment cases as well as between pre-treatment cases and controls. Using the inverse Simpson index to estimate diversity among IGH reads, no significant difference was observed between controls (37.32 ± 7.91—mean ± standard error of the mean) and T2 cases (55.49 ± 12.45), Bonferroni-adjusted p = 0.63 (S10 Fig). Contrary to our expectations, however, no significant difference was observed in aggregate between controls and T1 cases (28.69 ± 11.17) (p = 0.49), nor between T1 and T2 cases (n = 6; p = 0.94). Similarly, no significant differences were observed between all comparisons for IGK or IGL (S10 Fig). All other measured diversity indices are available in S11 Fig and S6 Table. Hierarchical clustering of diversity profiles as described by Greiff *et al*, did not suggest any clear separations by time or case status (S12 Fig) [42].

#### Principal component analysis

**Fig 13:**
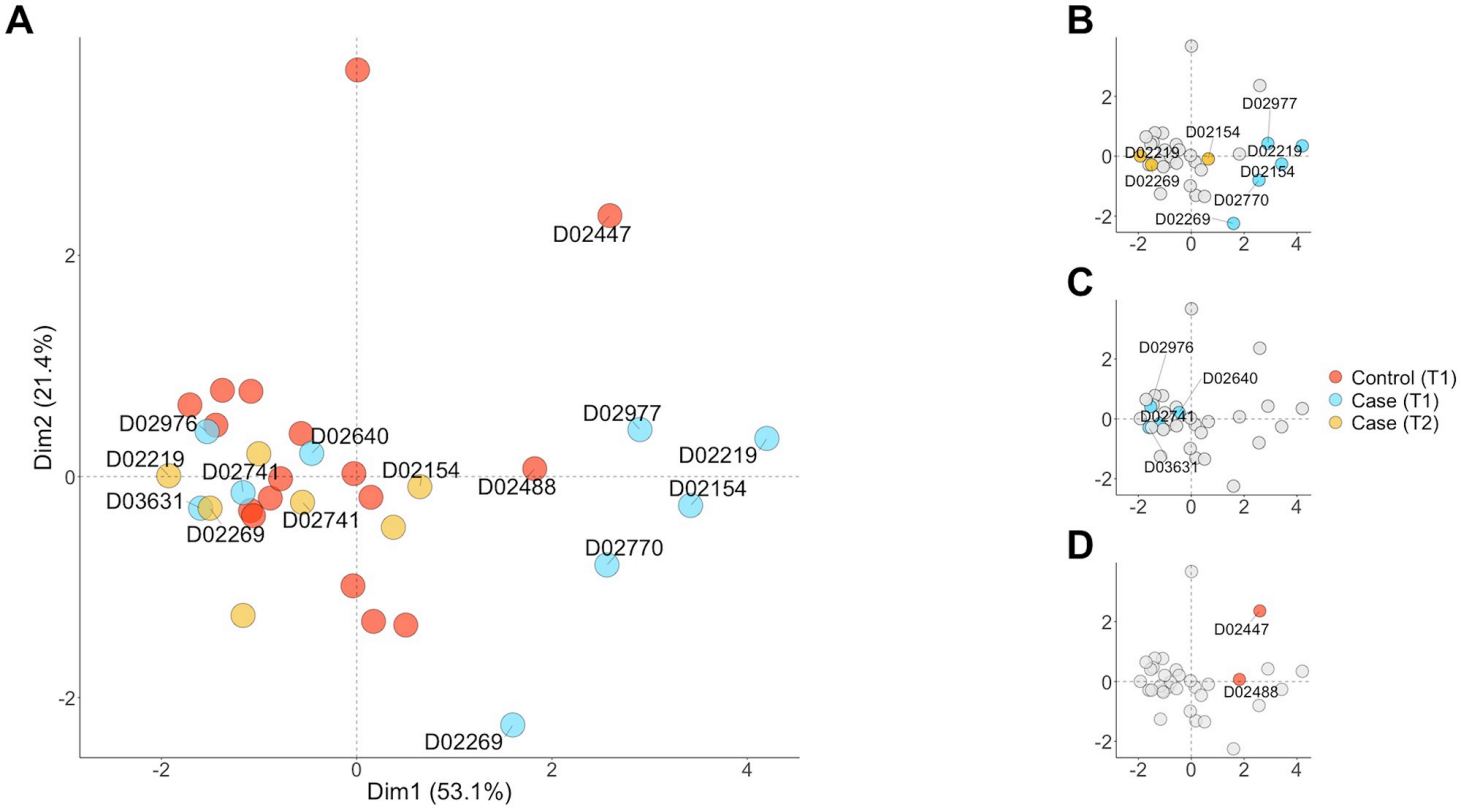
Principal component analysis for IGH. (A) All controls and T1 and T2 cases passing filtering criteria. (B) T1 cases are highlighted that follow expectation (i.e. appear clonal based on the frequency of a single clone at T1) with paired T2 clonal reduction for samples with both time points. (C) T1 cases that are grouped with the majority of controls. (D) Two controls that are grouped near clonal cases highlighted in panel A.

Because we did not observe significant differences in repertoire diversity between T1 and T2 cases or between T1 cases and controls at the group level (likely due to the large range in diversity estimates per group), we performed a principal components analysis (PCA) in order to look for smaller clusters of samples with similar patterns of clonality. The IGH PCA is depicted in Fig 13. Consistent with our findings in S10 Fig, we did not observe any group-level clusters by case status and time point for any of the three chains (PCA for IGK and IGL in S13 Fig).

However, we did observe a number of interesting groupings in the IGH data. There were five T1 cases clustered toward the right of the figure that were all highly clonal at this time point [mean proportion of most abundant clone = 0.753, 95% CI (0.565, 0.942)] (Fig 13B). All of these cases had T2 time points that moved to the left and group with the majority of the controls [0.104, 95% CI (-0.239, 0.448)]. There were also four T1 cases located toward the left of the figure with the majority of the controls and T2 cases and had low clonality at this time point [0.049, 95% CI (0.013, 0.085)] (Fig 13C). Finally, we observed two controls that were located near the highly clonal T1 cases [0.496, 95% CI (0.165, 0.827)] (Fig 13D).

#### Clonality vignettes

**Fig 14:**
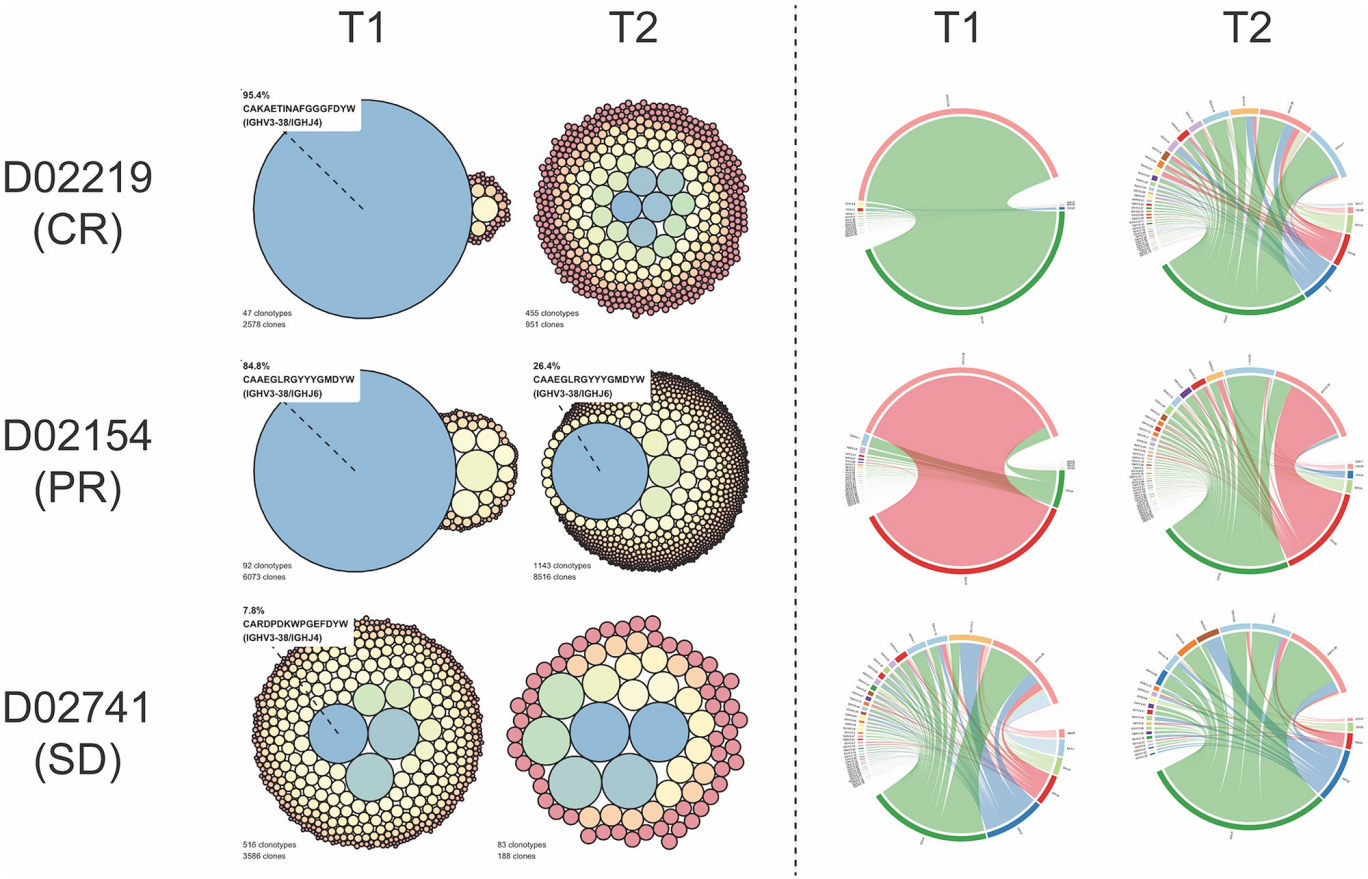
IGH clonality by clinical outcome. Circle size scaled to clonotype frequency (left) and VJ junction circos plots (right) at T1 and T2 for individual cases with complete remission (D02219), partial remission (D02154), and stable disease treated only with prednisone (D02741). Clonotype with highest frequency at T1 is labeled with CDR3 amino acid sequence and V and J genes. The same clonotype is labeled at T2 if identified within the repertoire.

Based on the PCA and clinical outcomes, we selected a trio of cases to interrogate further. To visually depict changes in clonality over time, we developed repertoire illustrations based on circle packing and VJ usage circos plots for three different clinical case examples: D02219 (complete remission—CR), D02154 (partial remission—PR), and D02741 (stable disease—SD and treated with prednisone only) (Fig 14). For D02219 (CR), there is a clear difference in the repertoire layouts between T1 and T2 with a single clonotype representing 95.4% of sequences at T1 that was not identified post-treatment. Similarly, VJ usage mimics this expansion and reduction with IGHV3-38—IGHJ4 pairing at 95.9% of the T1 repertoire reduced to 18.6% at T2. Moreover, there is a noticeable increase in varied V and J gene pairings at T2 compared to T1. These findings are consistent with the dog’s clinically observed complete remission. For D02154 (PR), the predominant clonotype at T1 (84.8%) was significantly reduced but still present at T2 (26.4%). The VJ usage plots also support this clinical outcome with the most abundant pairing of IGHV3-38—IGHJ6 (85.1%) reduced but still present at T2 (27.8%) and an overall more diverse set of VJ pairings as with D02219. For D02741 (SD), there are no major differences in the overall repertoires between T1 and T2. The clonotype with the highest frequency at T1 (7.8%) was not observed at T2. Additionally, the VJ pairings appear relatively diverse and similar pre- and post-treatment, suggesting there was not a major expansion of a single clonotype that could be identified.

## Discussion

This work represents the first major use of NGS to profile the complete BCR repertoire in healthy dogs and dogs undergoing treatment for intermediate-to-large B-cell lymphoma, a common canine hematopoietic neoplasm that we used as a model for B-cell clonality. By considering all of the chains in a comparatively unbiased and in-depth fashion, it has been possible to identify novel attributes of the healthy canine immune repertoire, and to begin to explore how these vary in a disease state. It has yielded novel insights into canine antibody light chains and built on previous work investigating the canine IGH locus and its expression. The work combines a wet lab protocol with an open source analysis pipeline in order to allow future researchers to apply them to their own sample sets and hypotheses. Beyond the protocol and healthy dog repertoire, this work also includes an initial investigation into the sequence diversity of B cells in the peripheral blood of patients with lymphoma. Typically, such experiments have focused on samples from lymph nodes or tumors directly, and so this investigation has taken a complementary approach to previous efforts.

### Protocol efficiency

The number of reads, and therefore the amount of data, that can be generated using NGS platforms is arguably their greatest strength. Particularly when applied to sequencing large and diverse repertoires, such as that of immunoglobulin transcripts. However, generating large amounts of data is not sufficient if only a limited fraction of it is usable. Within the dataset analyzed in this paper, the samples of several dogs were not included as they did not pass the filtering thresholds set. Of the dogs that were included, it was only a limited subset of their raw reads that passed filtering. However, this is in keeping with other approaches using 5’RACE, with one human study yielding sequences with identifiable V and J genes 2% of the time, and a more recent canine study using a similar approach unambiguously identifying the V and J in 13% of the sequenced pair end reads. What should be noted is that neither study used UMIs for error correction, and therefore did not have the perceived reduction in read count associated with collapsing reads based on shared UMIs, nor the filtering out of reads with insufficient UMIs [24, 57].

Overall, 5’RACE does offer the benefit of comparatively unbiased amplification, a strength compared to some PCR-based approaches. However, its inefficiencies can limit the capture of rare sequences. One way to address this would be through greater sequencing depth from a large, high quality sample. Alternatively, if a known rare sequence is being sought, such as in minimal residual disease detection, then specific primers can be used to capture those clonotypes in a way that 5’RACE might not be able to. However, when analyzing the overall repertoire 5’RACE remains an excellent tool for researchers to adapt to their experimental needs.

Given the large number of steps between the initial blood draw and the final processed reads, there are many points at which data abundance or quality can be compromised, but these also represent opportunities for future refinement. Ensuring high RNA quality and its faithful representation within the final library is of key importance. Given that the reverse transcription step occurs from a fixed point near the J-C junction, only transcripts with this region intact will be converted into substrate available for the next step. While careful use of blocking agents can reduce the incidence of the TSO priming in the middle of transcripts, there is no way to prevent template switching from truncated sequences. These events yield a library skewed towards coverage at the 3’ end of transcripts, and these shortened sequences make bioinformatic processing more challenging, for example by making it more difficult to definitively identify the V gene. Many filtering pipelines discard reads without a definitive V identified, and this reduction in data in turn can complicate other analyses.

In terms of bioinformatic filtering, this is linked both to the purpose of the experiment, and to confidence thresholds. For this work, only reads with unambiguously assigned V genes from functional transcripts were of interest. Furthermore, it was important to have confidence in the sequence identified, hence the MIG size of three was chosen as opposed to a MIG size of one for example where MIG-based error correction would not be possible. It should also be noted, that when reads are collapsed based on a shared UMI, this does not represent any data loss, unlike for example a read excluded because the V gene couldn’t be identified but represents an essential part of the data processing. Overall, it is at the discretion of the researchers using this pipeline to define the parameters that best suit their work.

Given the intent of the experiment, to interrogate the canine immunoglobulin repertoire, the parameters chosen were deemed to be necessary and the results yielded have been more than sufficient. Each sample that passed filtering has yielded thousands up to the hundreds of thousands of reads, which is in keeping with NGS experiments carried out on human and non-human primate samples [8, 51, 59]. Furthermore, the results presented here broadly agree with previous work using canine samples [1, 18, 24]. As such, these data can now serve as a benchmark against which future analyses can be compared.

### Bioinformatics

The publicly available pipeline (https://github.com/jonahcullen/Wrappar) enables researchers to analyze their own 5’RACE immunoglobulin or T-cell receptor sequencing data with minimal modification or bioinformatic knowledge. This pipeline utilizes a number of common bioinformatic tools for sequence error correction and assembly (MiGEC [14]), alignment and clonotype assembly (MiXCR [16]), and various repertoire analyses (e.g. CDR3 length, V and J gene usage, diversity estimates) (VDJtools [28]). Of note, it was observed that MiXCR assembled CDR3L clonotypes included three additional amino acids upstream of the conserved FR3 cysteine which required trimming prior to analysis (e.g. Seqlogo). Although we did not investigate this presumed error associated with the processing of canine BCR sequences, it could potentially be due to the application of this tool to a non-human species. We therefore encourage researchers to take care when applying this method to interrogate the IGL repertoire of non-human species. In addition to capturing and visualizing individual quality control data via MiGEC, log files from pre-processing, alignment, and clonotype assembly are parsed into plain text tables for quality control analyses across all included samples. Developing the pipeline with Snakemake enables scalability while enforcing reproducibility [26]. In order to run this pipeline efficiently, access to a high-performance computing (HPC) system using the Slurm scheduler is required. Usage with other commonly employed HPC schedulers (e.g. PBS, LSF, SGE) currently requires user-specific modification to the pipeline setup but this could be added in the future.

### Healthy dog repertoire

Overall, the repertoire features within the healthy canine samples match both what is expected and that which has been seen before, particularly for the heavy chain. What was novel, and therefore of most interest, was the detailed analysis of the light chain repertoires and unusual pattern of IGHV usage across samples, although the observed bimodal patterning of the latter was not found to be statistically significant.

#### Light chain repertoire analysis

Work that investigates immunoglobulin repertoires, regardless of species, generally focuses on just the heavy chain. It is easier to only process and analyze a single chain, and there is more variation in the CDR3H than any of the five other CDRs in an antibody sequence, so it has been seen as the most interesting to study. However, there are interesting facets to be considered within the light chain repertoire. Nearly all approved human therapeutic antibodies use a kappa chain and this can largely be traced back to their murine origin, given that the mouse is heavily biased towards kappa chain usage [56, 60]. Given that therapeutic antibodies are now being developed for dogs and cats, both of which are heavily lambda chain dominant, a better understanding of these chains and their features will be critical to that field, as well as to basic research more broadly.

Two things are of most note within the healthy light chain sequences analyzed here: biases in the CDR3L and biases in the CDR3K. Seventy IGLV genes were found to be expressed, as were all of the distinguishable IGLJs. This combinatorial diversity is reflected in the three most common CDR3Ls only accounting for a collective 10.0%, on par with the 7.9% for the top three CDR3Hs and far less than the cumulative 42.2% for CDR3Ks. Overall, therefore, CDR3Ls are diverse. However, looking at the CDR3Ls position by position tells a very different story, with pronounced biases at key positions. These biases could be attributable to structure, either stabilizing the loop itself by allowing better interfacing with the heavy chain CDRs, or potentially having features best suited to the types of antigens that dogs face. It should be noted that the dogs used here were ostensibly healthy and free from any therapeutic intervention that could skew their repertoire, and so these data represent the naive circulating repertoire. Further work is required to elucidate exactly what purpose these residues serve and what penalty is faced if they are changed.

In a similar vein, it is known that dog antibodies very rarely use the kappa chain. In mice, it has been determined that the IGK locus rearranges before the IGL locus, which may contribute to the kappa dominance in that species. The authors of this work are not aware of work in dogs that has formally analyzed the order of light chain rearrangements. Another contributing factor for the biased usage in each species is the number of genes available in the locus. This may have a ‘chicken and egg’ element to it. It is believed that the immunoglobulin loci have grown so large through the ‘birth and death’ model put forward by Masatoshi Nei and colleagues [61]. In this model, V genes are periodically duplicated within the locus and accumulate mutations. Those with beneficial mutations are retained and those without are lost.

The relevance here is that active and open chromatin is required for these duplication events. In this system it is not clear where cause and effect lie, whether the chromatin’s open and active status drives the increased duplication events at the locus, or the presence of more genes leads to more active chromatin. What can clearly be seen is that although IGK is used less frequently than IGL in dogs, when IGK is used there is a very pronounced bias to one of two near identical CDR3Ks. While the largely homogenous germline IGKV sequences and strong bias to a particular IGKV both likely contribute to this phenomenon, it doesn’t change the practical outcome of bias within the expressed antibody repertoire. Around half of CDR3Ks have positively charged residues at their tips (position 109), where nearly all CDR3Ls have a negatively charged residue near the top of their loops (position 108). This difference could influence factors such as CDR3H interactions or the ability to bind specific antigens. Identifying the exact impact of these biases requires further research, but this work does imply that biases in light chain expressed repertoires, either at individual points or across an entire CDR3, may serve important functions in the final expressed antibody.

#### Biased IGHV usage

Biased usage of certain V genes within a repertoire is a well documented feature, identified across multiple species in the literature. Furthermore, many of the reasons that underpin these biases have been described and include the age of the individual, chromatin state, health status, and sequence attributes of the V gene and its recombination signal sequence [11, 62–64]. Hwang and colleagues noted that, out of three dogs, one was dominant for IGHV3-38, one was dominant for IGHV4-1, and one was balanced between the two. They also observed that the ratio of IGHV3-38:IGHV4-1 was similar within an individual when comparing samples from bone marrow, lymph node, and spleen [24]. This work builds on that previous finding and shows that the strong negative correlation between these genes is seen across a larger number of individuals. That the observation is not statistically significant could well be because a subset of dogs are in fact balanced between the two genes.

What is of particular interest is that the V genes in question come from different IGHV families, and so are as distinct from each other as possible within the canine IGHV repertoire. It is not immediately clear from this dataset what underpins this difference: there is no obvious link to age, sex, breed, geography, or other defining trait of the dogs themselves. There was one case dog (D02640) that was initially dominant for IGHV4-1 at T1, but for IGHV3-38 at T2 (S7 Table). This could be explained by the preference for one gene being a fixed constant in an animal’s life, and that by chance that a clonal population developed in the alternate IGHV. Alternatively, individual animals could be dominant for either IGHV3-38 or IGHV4-1 in an alternating fashion over their lives and the preference for one over the other is transient. If preference does indeed alternate over time, then perhaps balanced dogs are in fact ones in transition between the states. IGHV4 family genes were found in humans to preferentially bind autoantigens and commensal bacteria [62]. Further work using the approach outlined in this paper could elucidate if the same holds true for dogs.

Ultimately, this experiment was not designed to answer the question of alternating V usage over the lifetime of a healthy animal, nor was it powered to investigate its potential relationship to factors such as breed or age. What this finding does underpin, is the value in working with individual, as opposed to pooled samples. It is possible that further work could identify other trends within canine V gene usage. For example, an approach that retains antibody pairing information such as single cell sequencing could elucidate whether there are light chains preferentially used with each of these dominant IGHVs. It also raises interesting questions about the applications to medicine. For example, do dogs biased for one or the other IGHV respond differently to immune challenges, and would immune-related therapies such as therapeutic antibodies behave differently in each class of dog?

### Clonality assessment of cases and controls

The inclusion criteria for cases in this dataset included the identification of a clonal population of B cells at the time of diagnosis, when T1 blood samples were drawn, which for one dog (D03631) was carried out by PARR and all other dogs by flow cytometry (FC). The expectation at the outset of this work was that: all control dogs would have no detectable clonality; T1 samples would be highly clonal; those dogs that responded well to chemotherapy would have a T2 repertoire reminiscent of control dogs; and those that had relapsed or that were otherwise not responding well would not only be clonal, but may well have the same dominant clonotype as in T1. Contrary to our expectations, these were not the uniform findings across all of the cases.

There were dogs that very well matched our hypothesis, and some of these are highlighted in the clonality vignettes (Fig 13). D02219 was highly clonal at T1 and appears much more diverse at T2, exactly as would be expected from a patient that has successfully entered remission by chemotherapy. D02154, a dog that achieved partial remission clinically, shows a noteworthy reduction in clonality, but the same dominant clonotype of T1 is present. Given that there are only two data points, it isn’t possible to definitively say whether this is a patient that went into remission and has since relapsed or, as we suspect given that it was only 6 weeks between T1 and T2, that they were only a partial responder to chemotherapy in the first instance. Finally, D02741 provided an example of a dog with stable disease over our 6-week window, based on comparative lymph node measurements at T1 vs T2. This is reflected in the balanced level of clonality, which is low, at both time points.

Within the PCA, Dim1 is largely driven by measures of clonality (inverse Simpson, Shannon-Wiener, Gini) and so samples that differ from each other along this axis can be primarily considered to differ in clonality. On the other hand, Dim2 is a factor of both the CDR3H length and the level of mutation from germline within each sequence (S14 Fig). Considering the other cases shown in Fig 13, there are the five that had the expected clonality at T1 or T2 (Fig 13B). D02219 and D02154 were in the vignette and so have been discussed above, and D02269 had a similar reduction in clonality across the two timepoints. D02770 unfortunately died before T2 but seemed to be quite clonal at T1. D02977 did not yield enough reads at T2 to be included in the analyses but was clustered with the other clonal samples at T1.

However, there were a number of dogs that didn’t follow this trend (Fig 13C), including D02741, which was covered in the vignette. The others, D02976, D02640, and D03631 were all grouped with the control dogs and so were not clonal as expected. Given that it is unlikely that the 5 ‘RACE method used was not sensitive enough to detect clonality, or that there was some other bias in the protocol to prevent the detection of clonality in a processed sample, it would imply that the sample itself did not contain RNA from clonal B cells. This is actually in keeping with previous work. Aresu and colleagues looked at 14 dogs with diffuse large B-cell lymphoma and in all cases could identify clonality with both PARR and FC in lymph node samples taken at pretreatment staging (equivalent to our T1). However, only half of the peripheral blood samples drawn at the same time were positive on both PARR and FC. Of the remaining seven, four appeared negative on both PARR and FC for lymphoma, in spite of the positive lymph node diagnosis [65]. We therefore speculate that the reason we were not able to identify clonality in these samples was due to the choice of peripheral blood as the sample, rather than a lymph node aspirate or biopsy. Future work using this protocol on lymph node aspirates from dogs with B cell lymphoma would help establish whether that is indeed the case.

Finally, there is the question of the two control dogs with apparent clonality, D02447 and D02488. These were ostensibly healthy animals, with no diagnosis that could explain the perceived lack of diversity in their samples. Closer analysis reveals that D02447 had longer CDR3Hs (weighted mean length of 19.52) without an especially high mutational load compared to other controls. This distribution of the factors that contribute to Dim2 largely account for its position further along Dim1, but in turn raise the question as to what the explanation is for the large CDR3Hs with low mutational load. Lower mutational load generally implies B cells earlier in development, as they have had less chance to undergo somatic hypermutation. That the overall sample was weighted towards these is either a factor of sample preparation or that there had been a recent proliferation in B cells and the blood was drawn early in the affinity maturation process. For D02488 there is less of note that explains its positioning, other than the fact that its measures of clonality were slightly higher than its control peers. Other than lymphoma, clonal B cell populations can be identified in response to various bacterial infections, as well as parasitic infections caused by heartworm and leishmaniasis. Given that both dogs were not showing noticeable signs of disease, it is most likely that they were facing a mild immune challenge—enough to slightly reduce their clonality but not so much as to be symptomatic [66–69].

In summary, this work has developed and applied a novel protocol and analysis pipeline to the canine BCR repertoire. It has built on what is known for canine IGH expression and added new insights to the canine antibody light chains. It has also demonstrated its potential to compare individuals and disease states and can serve as a useful basis for future work in healthy dogs or those with conditions affecting their B cell receptor repertoire. Subsequent work could, in particular, delve further into what influences IGHV usage within and across individuals, and the varying levels of clonality in the various immune compartments of a patient over the course of disease and treatment. Adapting the protocol to single-cell analysis would unlock the ability to consider paired antibody sequences in a high-throughput fashion and would likely elucidate the relationships of the features and biases of each of the heavy and light chains.

## Supporting information

S1 Fig Pre-processed sequence read counts. Count of sequencing reads for enrolled cases (n = 18) and controls (n = 25) prior to processing.
(TIFF)

S2 Fig VJ reads per UMI size. Proportions of sample-time total VJ reads represented by 3–20 reads per UMI.
(TIFF)

S3 Fig Percent of reads not assigned to clonotype by count. Percentage of VJ reads not assigned to a clonotype by the count (log scale) of VJ reads for each chain. Green box represents included chains with greater than 1000 reads and a maximum of 95% of reads not assigned to a clonotype by MiXCR.
(TIFF)

S4 Fig Attrition from raw sequences through total clones. Decrease (log scale) in sequencing reads starting with raw input to reads containing master barcode (e.g. IGM, IGG, IGA, IGE, IGK, IGL), number of reads in assembled consensus sequences, total consensus sequences, VJ reads (potential clones), VJ reads successfully aligned again IMGT database, and total number of clones. Note the decrease from “Reads in assembled consensuses” to “Assembled consensuses” is not a filtering but a collapse.
(TIFF)

S5 Fig Circos plots of paired VJ usage by chain. Around the outer edge of each plot are the V and J genes from each of the (A) IGH, (B) IGK, and (C) IGL loci. The thickness of the band joining any pair of V and J genes on the plot represents the unweighted proportion of reads that used that combination of V and J genes.
(TIFF)

S6 Fig CDR3 properties by chain. Reported properties at each CDR3 position, including hydropathy, size, chemical class, charge, hydrogen donor or acceptor class, and polarity were calculated based upon the proportion of amino acids at each position averaged across included samples using standard biochemical properties [34]. Only CDR3s of the most common length for (IGH = 13, IGK = 9, and IGL = 11) were considered.
(TIFF)

S7 Fig V gene usage profile heatmaps. For each chain, V gene usage profiles are clustered using VDJtools’ “CalcSegmentUsage” method to compare both T1 cases and controls (left), and T1 and T2 cases (right) [28].
(TIF)

S8 Fig J gene usage profile heatmaps. For each chain, J gene usage profiles are clustered using VDJtools’ “CalcSegmentUsage” method to compare both T1 cases and controls (left), and T1 and T2 cases (right) [28].
(TIF)

S9 Fig Clonotype repertoire overlap for IGH, IGK, and IGL. Clonotypes shared across time points are plotted for three cases (D02154, D02269, and D02640) where all three chains passed filtering criteria. The fitted regression equation and *R*^*2*^ are included. Jitter is applied to the data points. Dashed line (through the origin with slope 1) represents perfect concordance between T1 and T2 clonotypes (*i*.*e*. clonotypes observed at the same frequency in T1 and T2). Deviations from the dashed line indicate differences in clonotype frequency. VDJtools’ “OverlapPair” was used to identify shared clonotypes based on CDR3 amino acid sequence and V and J gene [28].
(TIFF)

S10 Fig Inverse Simpson index for IGH, IGK, and IGL. Inverse Simpson index as calculated by VDJtools “CalcDiversityStats” method for controls, and T1 and T2 cases across all chains [28]. Samples with clone size less than 100 were excluded in order to increase the accuracy of the resampling estimates. Refer to Table 1 for samples with paired comparisons between T1 and T2 (6 IGH, 4 IGK, and 7 IGL).
(TIFF)

S11 Fig All calculated diversity estimates for IGH, IGK, and IGL. Control, T1 and T2 case diversity estimates of observed diversity, Chao1, Efron-Thisted, normalized Shannon-Wiener, and d50 by VDJtools’ “CalcDiversityStats” method [28]. Number of clones and the Gini index (calculated with the R package *ineq* [46]) are also included. D02219 had only two reported IGL clonotypes at T1 thus precluding the ability to calculate the normalized Shannon-Wiener index by VDJtools.
(TIFF)

S12 Fig Cluster heatmap of Euclidean distances between diversity profiles. Euclidean distances were calculated pairwise between diversity profiles, followed by complete-linkage clustering, and visualization with *ComplexHeatmap* [44]. Case status and time point are included as annotations by color for (A) IGH, (B) IGK, and (C) IGL.
(TIFF)

S13 Fig Principal component analysis for IGK and IGL. (A) IGK. (B) IGL. The normalized Shannon-Wiener index could not be calculated by VDJtools for D02219 at T1 and thus the single time point was removed.
(TIFF)

S14 Fig Principal component variable and contribution plots for IGH, IGK, and IGL. Percent contribution of each variable along the first and second principal components.
(TIFF)

S1 Table 5’ RACE protocol primers. (XLSX)

S2 Table Clinical data for enrolled cases. (XLSX)

S3 Table Clinical data for enrolled controls. (XLSX)

S4 Table Excluded chains. Chains with less than 1000 reads or greater than 95% of reads not assigned to clonotype (in grey) that were excluded for each sample and time point.
(XLSX)

S5 Table Jaccard indices. Jaccard index (calculated by VDJtools’ “OverlapPair” method) for three cases (D02154, D02269, and D02640) where all three chains passed filtering criteria [28].
(XLSX)

S6 Table Mean diversity estimates and standard errors. (XLSX)

S7 Table Proportional usage of IGHV3-38 and IGHV4-1 in cases at T1 and T2. (XLSX)

## References

[1] Steiniger SCJ, Dunkle WE, Bammert GF, Wilson TL, Krishnan A, Dunham SA, et al. Fundamental characteristics of the expressed immunoglobulin VH and VL repertoire in different canine breeds in comparison with those of humans and mice. Mol Immunol. 2014;59: 71–78. doi: 10.1016/j.molimm.2014.01.010 24509215

[2] Martin J, Ponstingl H, Lefranc M-P, Archer J, Sargan D, Bradley A. Comprehensive annotation and evolutionary insights into the canine (Canis lupus familiaris) antigen receptor loci. Immunogenetics. 2018;70: 223–236. doi: 10.1007/s00251-017-1028-0 28924718 PMC5871656

[3] Glanville J, Zhai W, Berka J, Telman D, Huerta G, Mehta GR, et al. Precise determination of the diversity of a combinatorial antibody library gives insight into the human immunoglobulin repertoire. Proc Natl Acad Sci U S A. 2009;106: 20216–20221. doi: 10.1073/pnas.0909775106 19875695 PMC2787155

[4] Greiff V, Menzel U, Miho E, Weber C, Riedel R, Cook S, et al. Systems Analysis Reveals High Genetic and Antigen-Driven Predetermination of Antibody Repertoires throughout B Cell Development. Cell Rep. 2017;19: 1467–1478. doi: 10.1016/j.celrep.2017.04.054 28514665

[5] Kirsch I, Vignali M, Robins H. T-cell receptor profiling in cancer. Mol Oncol. 2015;9: 2063–2070. doi: 10.1016/j.molonc.2015.09.003 26404496 PMC5528728

[6] Gazzola A, Mannu C, Rossi M, Laginestra MA, Sapienza MR, Fuligni F, et al. The evolution of clonality testing in the diagnosis and monitoring of hematological malignancies. Ther Adv Hematol. 2014;5: 35–47. doi: 10.1177/2040620713519729 24688753 PMC3949299

[7] Blachly JS, Ruppert AS, Zhao W, Long S, Flynn J, Flinn I, et al. Immunoglobulin transcript sequence and somatic hypermutation computation from unselected RNA-seq reads in chronic lymphocytic leukemia. Proc Natl Acad Sci U S A. 2015;112: 4322–4327. doi: 10.1073/pnas.1503587112 25787252 PMC4394264

[8] DeKosky BJ, Lungu OI, Park D, Johnson EL, Charab W, Chrysostomou C, et al. Large-scale sequence and structural comparisons of human naive and antigen-experienced antibody repertoires. Proc Natl Acad Sci U S A. 2016;113: E2636–2645. doi: 10.1073/pnas.1525510113 27114511 PMC4868480

[9] Mamedov IZ, Britanova OV, Zvyagin IV, Turchaninova MA, Bolotin DA, Putintseva EV, et al. Preparing Unbiased T-Cell Receptor and Antibody cDNA Libraries for the Deep Next Generation Sequencing Profiling. Front Immunol. 2013;4. doi: 10.3389/fimmu.2013.00456 24391640 PMC3870325

[10] Freeman JD, Warren RL, Webb JR, Nelson BH, Holt RA. Profiling the T-cell receptor beta-chain repertoire by massively parallel sequencing. Genome Res. 2009;19: 1817–1824. doi: 10.1101/gr.092924.109 19541912 PMC2765271

[11] Choi NM, Loguercio S, Verma-Gaur J, Degner SC, Torkamani A, Su AI, et al. Deep sequencing of the murine IgH repertoire reveals complex regulation of nonrandom V gene rearrangement frequencies. J Immunol Baltim Md 1950. 2013;191: 2393–2402. doi: 10.4049/jimmunol.1301279 23898036 PMC3778908

[12] Kivioja T, Vähärautio A, Karlsson K, Bonke M, Enge M, Linnarsson S, et al. Counting absolute numbers of molecules using unique molecular identifiers. Nat Methods. 2012;9: 72–74. doi: 10.1038/nmeth.1778 22101854

[13] Turchaninova MA, Davydov A, Britanova OV, Shugay M, Bikos V, Egorov ES, et al. High-quality full-length immunoglobulin profiling with unique molecular barcoding. Nat Protoc. 2016;11: 1599–1616. doi: 10.1038/nprot.2016.093 27490633

[14] Shugay M, Britanova OV, Merzlyak EM, Turchaninova MA, Mamedov IZ, Tuganbaev TR, et al. Towards error-free profiling of immune repertoires. Nat Methods. 2014;11: 653–655. doi: 10.1038/nmeth.2960 24793455

[15] Khan TA, Friedensohn S, de Vries ARG, Straszewski J, Ruscheweyh H-J, Reddy ST. Accurate and predictive antibody repertoire profiling by molecular amplification fingerprinting. Sci Adv. 2016;2: e1501371. doi: 10.1126/sciadv.1501371 26998518 PMC4795664

[16] Bolotin DA, Poslavsky S, Mitrophanov I, Shugay M, Mamedov IZ, Putintseva EV, et al. MiXCR: software for comprehensive adaptive immunity profiling. Nat Methods. 2015;12: 380–381. doi: 10.1038/nmeth.3364 25924071

[17] Bolotin DA, Poslavsky S, Davydov AN, Frenkel FE, Fanchi L, Zolotareva OI, et al. Antigen receptor repertoire profiling from RNA-seq data. Nat Biotechnol. 2017;35: 908–911. doi: 10.1038/nbt.3979 29020005 PMC6169298

[18] Bao Y, Guo Y, Xiao S, Zhao Z. Molecular characterization of the VH repertoire in Canis familiaris. Vet Immunol Immunopathol. 2010;137: 64–75. doi: 10.1016/j.vetimm.2010.04.011 20483487

[19] Sato M, Yamazaki J, Goto-Koshino Y, Setoguchi A, Takahashi M, Baba K, et al. Minimal residual disease in canine lymphoma: An objective marker to assess tumour cell burden in remission. Vet J Lond Engl 1997. 2016;215: 38–42. doi: 10.1016/j.tvjl.2016.05.012 27339366

[20] Rütgen BC, König R, Hammer SE, Groiss S, Saalmüller A, Schwendenwein I. Composition of lymphocyte subpopulations in normal canine lymph nodes. Vet Clin Pathol. 2015;44: 58–69. doi: 10.1111/vcp.12221 25512102

[21] Chen H-W, Small GW, Motsinger-Reif A, Suter SE, Richards KL. VH1-44 gene usage defines a subset of canine B-cell lymphomas associated with better patient survival. Vet Immunol Immunopathol. 2014;157: 125–130. doi: 10.1016/j.vetimm.2013.10.020 24332568 PMC3923267

[22] Hwang M-H, Darzentas N, Bienzle D, Moore PF, Guscetti F, Morrison J, et al. A review of canine B cell clonality assays and primer set optimization using large-scale repertoire data. Vet Immunol Immunopathol. 2019;209: 45–52. doi: 10.1016/j.vetimm.2019.01.002 30885305

[23] Takanosu M, Okada K, Kagawa Y. PCR-based clonality analysis of antigen receptor gene rearrangements in canine cutaneous plasmacytoma. Vet J Lond Engl 1997. 2018;241: 31–37. doi: 10.1016/j.tvjl.2018.09.010 30340657

[24] Hwang M-H, Darzentas N, Bienzle D, Moore PF, Morrison J, Keller SM. Characterization of the canine immunoglobulin heavy chain repertoire by next generation sequencing. Vet Immunol Immunopathol. 2018;202: 181–190. doi: 10.1016/j.vetimm.2018.07.002 30078594

[25] Lee GKC, Bienzle D, Keller SM, Hwang M-H, Darzentas N, Chang H, et al. Use of immune repertoire sequencing to resolve discordant microscopic and immunochemical findings in a case of T cell-rich large B cell lymphoma in a young dog. BMC Vet Res. 2021;17: 85. doi: 10.1186/s12917-021-02783-3 33602231 PMC7890612

[26] Koster J, Rahmann S. Snakemake—a scalable bioinformatics workflow engine. Bioinformatics. 2012;28: 2520–2522. doi: 10.1093/bioinformatics/bts480 22908215

[27] Lefranc M-P, Giudicelli V, Ginestoux C, Jabado-Michaloud J, Folch G, Bellahcene F, et al. IMGT, the international ImMunoGeneTics information system. Nucleic Acids Res. 2009;37: D1006–1012. doi: 10.1093/nar/gkn838 18978023 PMC2686541

[28] Shugay M, Bagaev DV, Turchaninova MA, Bolotin DA, Britanova OV, Putintseva EV, et al. VDJtools: Unifying Post-analysis of T Cell Receptor Repertoires. Gardner PP, editor. PLOS Comput Biol. 2015;11: e1004503. doi: 10.1371/journal.pcbi.1004503 26606115 PMC4659587

[29] Ye J, Ma N, Madden TL, Ostell JM. IgBLAST: an immunoglobulin variable domain sequence analysis tool. Nucleic Acids Res. 2013;41: W34–40. doi: 10.1093/nar/gkt382 23671333 PMC3692102

[30] Gupta NT, Vander Heiden JA, Uduman M, Gadala-Maria D, Yaari G, Kleinstein SH. Change-O: a toolkit for analyzing large-scale B cell immunoglobulin repertoire sequencing data. Bioinforma Oxf Engl. 2015;31: 3356–3358. doi: 10.1093/bioinformatics/btv359 26069265 PMC4793929

[31] Galson JD, Schaetzle S, Bashford-Rogers RJM, Raybould MIJ, Kovaltsuk A, Kilpatrick GJ, et al. Deep Sequencing of B Cell Receptor Repertoires From COVID-19 Patients Reveals Strong Convergent Immune Signatures. Front Immunol. 2020;11: 3283. doi: 10.3389/fimmu.2020.605170 33384691 PMC7769841

[32] Brunson JC, Read QD. ggalluvial: Alluvial Plots in “ggplot2.” 2020. https://CRAN.R-project.org/package=ggalluvial.

[33] Vadim Nazarov, immunarch.bot, Eugene Rumynskiy. immunomind/immunarch: 0.6.5: Basic single-cell support. Zenodo; 2020. 10.5281/zenodo.3893991.

[34] Pommié C, Levadoux S, Sabatier R, Lefranc G, Lefranc M-P. IMGT standardized criteria for statistical analysis of immunoglobulin V-REGION amino acid properties. J Mol Recognit JMR. 2004;17: 17–32. doi: 10.1002/jmr.647 14872534

[35] Wagih O. ggseqlogo: a versatile R package for drawing sequence logos. Bioinforma Oxf Engl. 2017;33: 3645–3647. doi: 10.1093/bioinformatics/btx469 29036507

[36] Wagih O. ggseqlogo: A “ggplot2” Extension for Drawing Publication-Ready Sequence Logos. 2017. https://CRAN.R-project.org/package=ggseqlogo.

[37] Schneider TD, Stephens RM. Sequence logos: a new way to display consensus sequences. Nucleic Acids Res. 1990;18: 6097–6100. doi: 10.1093/nar/18.20.6097 2172928 PMC332411

[38] Chu ND, Bi HS, Emerson RO, Sherwood AM, Birnbaum ME, Robins HS, et al. Longitudinal immunosequencing in healthy people reveals persistent T cell receptors rich in highly public receptors. BMC Immunol. 2019;20: 19. doi: 10.1186/s12865-019-0300-5 31226930 PMC6588944

[39] Tanno H, Gould TM, McDaniel JR, Cao W, Tanno Y, Durrett RE, et al. Determinants governing T cell receptor α/β-chain pairing in repertoire formation of identical twins. Proc Natl Acad Sci. 2020;117: 532–540. doi: 10.1073/pnas.1915008117 31879353 PMC6955297

[40] Dhar A, Davidsen K, Iv FAM, Minin VN. Predicting B cell receptor substitution profiles using public repertoire data. PLOS Comput Biol. 2018;14: e1006388. doi: 10.1371/journal.pcbi.1006388 30332400 PMC6205660

[41] Werner L, Lee YN, Rechavi E, Lev A, Yerushalmi B, Ling G, et al. Alterations in T and B Cell Receptor Repertoires Patterns in Patients With IL10 Signaling Defects and History of Infantile-Onset IBD. Front Immunol. 2020;11: 109. doi: 10.3389/fimmu.2020.00109 32117262 PMC7017840

[42] Greiff V, Bhat P, Cook SC, Menzel U, Kang W, Reddy ST. A bioinformatic framework for immune repertoire diversity profiling enables detection of immunological status. Genome Med. 2015;7: 49. doi: 10.1186/s13073-015-0169-8 26140055 PMC4489130

[43] Oksanen J, Blanchet FG, Friendly M, Kindt R, Legendre P, McGlinn D, et al. vegan: Community Ecology Package. 2020. https://CRAN.R-project.org/package=vegan.

[44] Gu Z, Eils R, Schlesner M. Complex heatmaps reveal patterns and correlations in multidimensional genomic data. Bioinforma Oxf Engl. 2016;32: 2847–2849. doi: 10.1093/bioinformatics/btw313 27207943

[45] Galson JD, Clutterbuck EA, Trück J, Ramasamy MN, Münz M, Fowler A, et al. BCR repertoire sequencing: different patterns of B cell activation after two Meningococcal vaccines. Immunol Cell Biol. 2015;93: 885–895. doi: 10.1038/icb.2015.57 25976772 PMC4551417

[46] Zeileis A, Kleiber C. ineq: Measuring Inequality, Concentration, and Poverty. 2014. https://CRAN.R-project.org/package=ineq.

[47] Zhigalova EA, Izosimova AI, Yuzhakova DV, Volchkova LN, Shagina IA, Turchaninova MA, et al. RNA-Seq-Based TCR Profiling Reveals Persistently Increased Intratumoral Clonality in Responders to Anti-PD-1 Therapy. Front Oncol. 2020;10: 385. doi: 10.3389/fonc.2020.00385 32411589 PMC7199218

[48] Morris EK, Caruso T, Buscot F, Fischer M, Hancock C, Maier TS, et al. Choosing and using diversity indices: insights for ecological applications from the German Biodiversity Exploratories. Ecol Evol. 2014;4: 3514–3524. doi: 10.1002/ece3.1155 25478144 PMC4224527

[49] Yaari G, Kleinstein SH. Practical guidelines for B-cell receptor repertoire sequencing analysis. Genome Med. 2015;7: 121. doi: 10.1186/s13073-015-0243-2 26589402 PMC4654805

[50] Kitaura K, Shini T, Matsutani T, Suzuki R. A new high-throughput sequencing method for determining diversity and similarity of T cell receptor (TCR) α and β repertoires and identifying potential new invariant TCR α chains. BMC Immunol. 2016;17: 38. doi: 10.1186/s12865-016-0177-5 27729009 PMC5059964

[51] Bashford-Rogers RJM, Palser AL, Huntly BJ, Rance R, Vassiliou GS, Follows GA, et al. Network properties derived from deep sequencing of human B-cell receptor repertoires delineate B-cell populations. Genome Res. 2013;23: 1874–1884. doi: 10.1101/gr.154815.113 23742949 PMC3814887

[52] Krishna C, Chowell D, Gönen M, Elhanati Y, Chan TA. Genetic and environmental determinants of human TCR repertoire diversity. Immun Ageing A. 2020;17: 26. doi: 10.1186/s12979-020-00195-9 32944053 PMC7487954

[53] Kassambara A, Mundt F. factoextra: Extract and Visualize the Results of Multivariate Data Analyses. 2020. https://CRAN.R-project.org/package=factoextra.

[54] Wang W, Wang H, Dai G, Wang H. Visualization of large hierarchical data by circle packing. Proceedings of the SIGCHI Conference on Human Factors in Computing Systems. New York, NY, USA: Association for Computing Machinery; 2006. pp. 517–520. doi: 10.1145/1124772.1124851

[55] Bedward M, Eppstein D, Menzel P. packcircles: Circle Packing. 2020. https://CRAN.R-project.org/package=packcircles.

[56] Arun SS, Breuer W, Hermanns W. Immunohistochemical examination of light-chain expression (lambda/kappa ratio) in canine, feline, equine, bovine and porcine plasma cells. Zentralbl Veterinarmed A. 1996;43: 573–576. doi: 10.1111/j.1439-0442.1996.tb00489.x 8968166

[57] Arnaout R, Lee W, Cahill P, Honan T, Sparrow T, Weiand M, et al. High-resolution description of antibody heavy-chain repertoires in humans. PloS One. 2011;6: e22365. doi: 10.1371/journal.pone.0022365 21829618 PMC3150326

[58] Zemlin M, Klinger M, Link J, Zemlin C, Bauer K, Engler JA, et al. Expressed murine and human CDR-H3 intervals of equal length exhibit distinct repertoires that differ in their amino acid composition and predicted range of structures. J Mol Biol. 2003;334: 733–749. doi: 10.1016/j.jmb.2003.10.007 14636599

[59] Sundling C, Zhang Z, Phad GE, Sheng Z, Wang Y, Mascola JR, et al. Single-cell and deep sequencing of IgG-switched macaque B cells reveal a diverse Ig repertoire following immunization. J Immunol Baltim Md 1950. 2014;192: 3637–3644. doi: 10.4049/jimmunol.1303334 24623130 PMC3993955

[60] Homepage of The Antibody Society. In: The Antibody Society [Internet]. [cited 18 Dec 2021]. https://www.antibodysociety.org/home/.

[61] Nei M, Gu X, Sitnikova T. Evolution by the birth-and-death process in multigene families of the vertebrate immune system. Proc Natl Acad Sci. 1997;94: 7799–7806. doi: 10.1073/pnas.94.15.7799 9223266 PMC33709

[62] Bashford-Rogers RJM, Bergamaschi L, McKinney EF, Pombal DC, Mescia F, Lee JC, et al. Analysis of the B cell receptor repertoire in six immune-mediated diseases. Nature. 2019;574: 122–126. doi: 10.1038/s41586-019-1595-3 31554970 PMC6795535

[63] Bolland DJ, Koohy H, Wood AL, Matheson LS, Krueger F, Stubbington MJT, et al. Two Mutually Exclusive Local Chromatin States Drive Efficient V(D)J Recombination. Cell Rep. 2016;15: 2475–2487. doi: 10.1016/j.celrep.2016.05.020 27264181 PMC4914699

[64] Matheson LS, Bolland DJ, Chovanec P, Krueger F, Andrews S, Koohy H, et al. Local Chromatin Features Including PU.1 and IKAROS Binding and H3K4 Methylation Shape the Repertoire of Immunoglobulin Kappa Genes Chosen for V(D)J Recombination. Front Immunol. 2017;8: 1550. doi: 10.3389/fimmu.2017.01550 29204143 PMC5698286

[65] Aresu L, Aricò A, Ferraresso S, Martini V, Comazzi S, Riondato F, et al. Minimal residual disease detection by flow cytometry and PARR in lymph node, peripheral blood and bone marrow, following treatment of dogs with diffuse large B-cell lymphoma. Vet J Lond Engl 1997. 2014;200: 318–324. doi: 10.1016/j.tvjl.2014.03.006 24698669

[66] de Caprariis D, Sasanelli M, Paradies P, Otranto D, Lia R. Monoclonal gammopathy associated with heartworm disease in a dog. J Am Anim Hosp Assoc. 2009;45: 296–300. doi: 10.5326/0450296 19887388

[67] Antognoni MT, Birettoni F, Miglio A, Lalli P, Porciello F, Mangili Pecci V. Monoclonal gammopathy associated with multiple myeloma and visceral leishmaniasis in the dog: A comparison of two cases. Vet Res Commun. 2010;34: 97–101. doi: 10.1007/s11259-010-9365-6 20461463

[68] Harrus S, Ofri R, Aizenberg I, Waner T. Acute blindness associated with monoclonal gammopathy induced by Ehrlichia canis infection. Vet Parasitol. 1998;78: 155–160. doi: 10.1016/s0304-4017(98)00132-0 9735920

[69] Benchekroun G, Desmyter A, Hidalgo A, Boulouis H-J, Gomes E, Garnier F, et al. Primary Hyperparathyroidism and Monoclonal Gammopathy in a Dog. J Vet Intern Med. 2009;23: 211–214. doi: 10.1111/j.1939-1676.2008.0223.x 19175743

