# Supplementary figures and images for: Development and application of a next-generation sequencing protocol and bioinformatics pipeline for the comprehensive analysis of the canine immunoglobulin repertoire
Source: PLoS One. 2022 Jul 8;17(7):e0270710. doi: 10.1371/journal.pone.0270710 (PMC9269486; doi:10.1371/journal.pone.0270710)

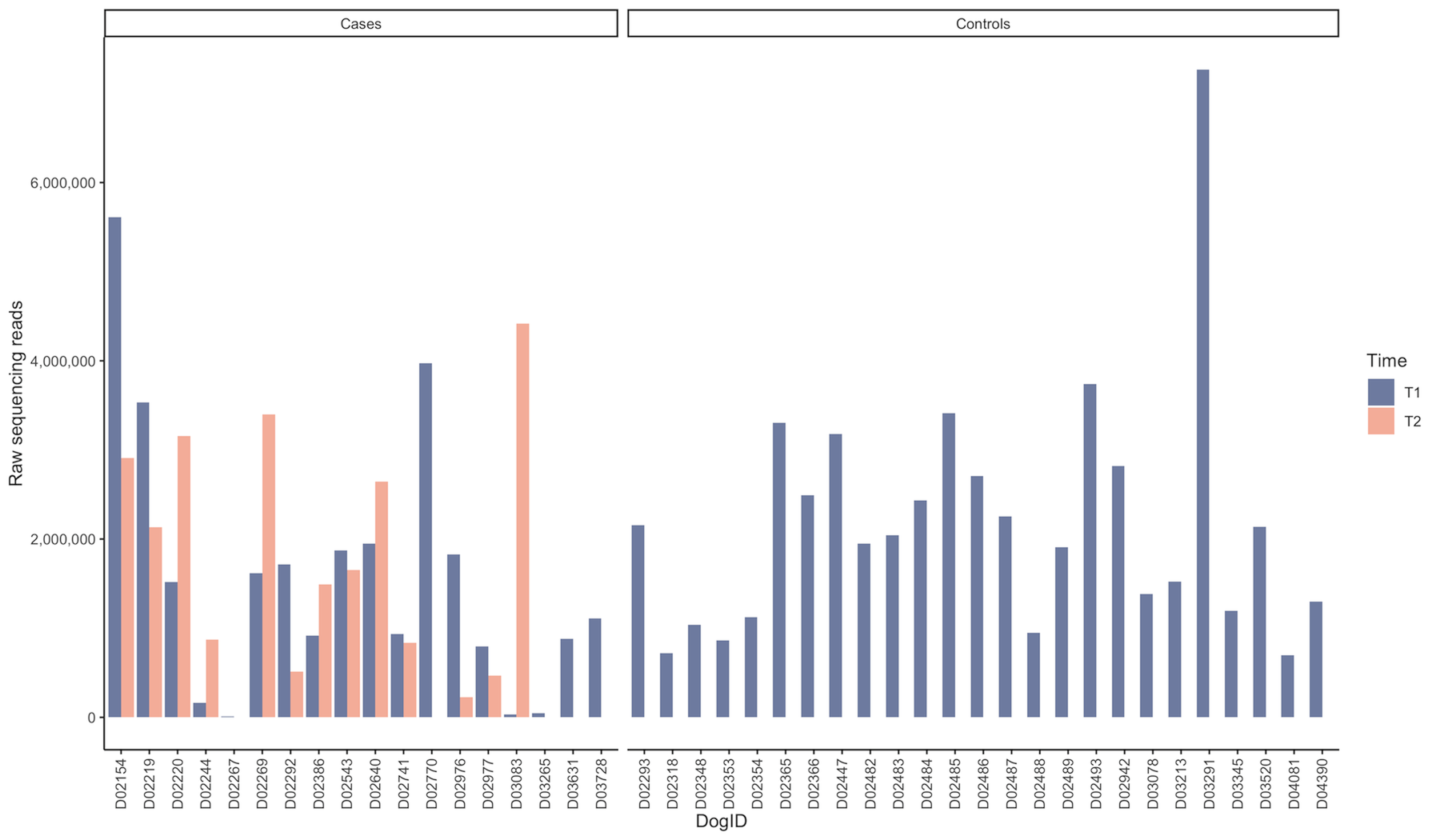

Supplement: S1 Fig — Count of sequencing reads for enrolled cases (n = 18) and controls (n = 25) prior to processing. (TIFF) [file pone.0270710.s001.tiff]

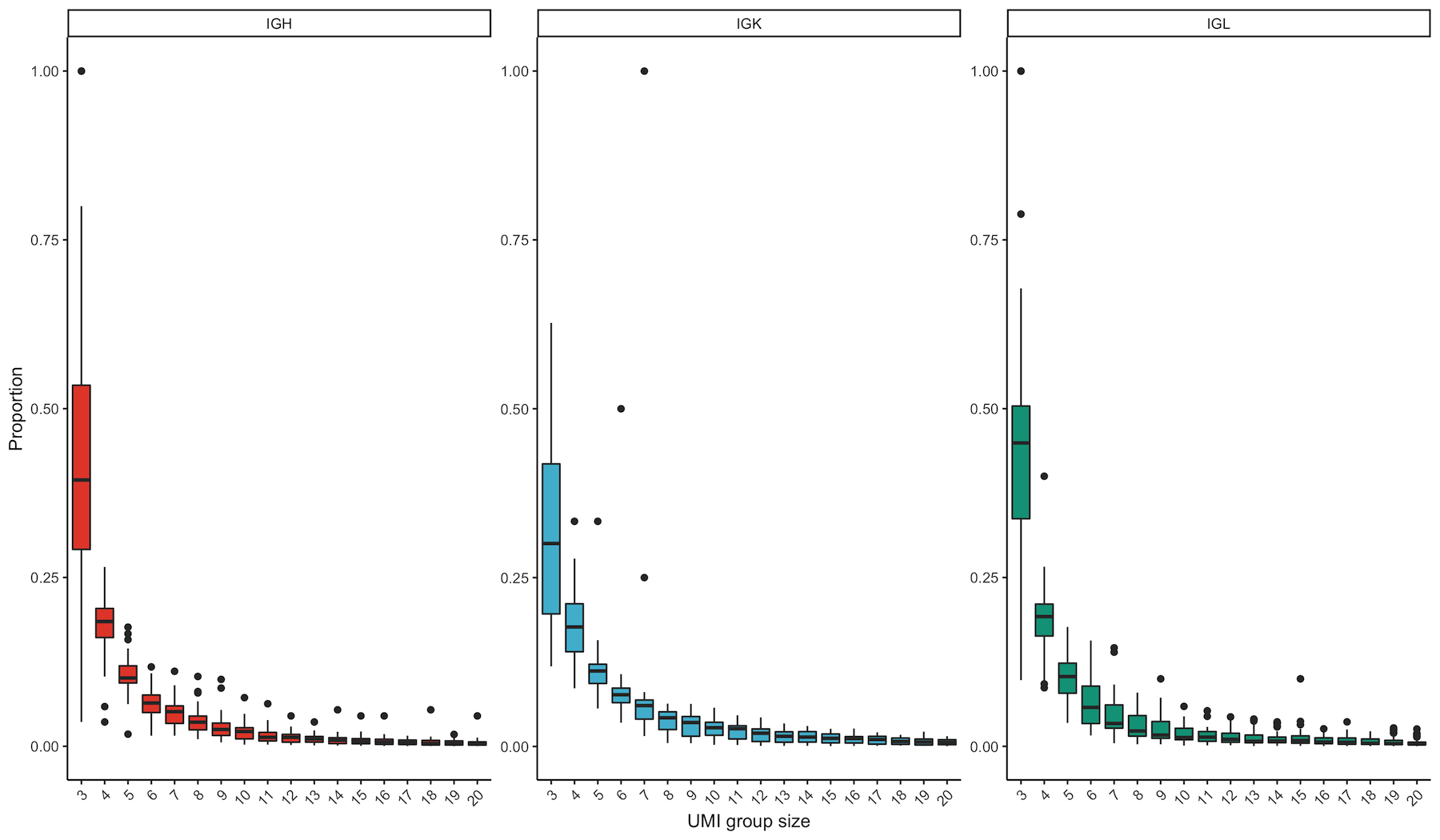

Supplement: S2 Fig — Proportions of sample-time total VJ reads represented by 3–20 reads per UMI. (TIFF) [file pone.0270710.s002.tiff]

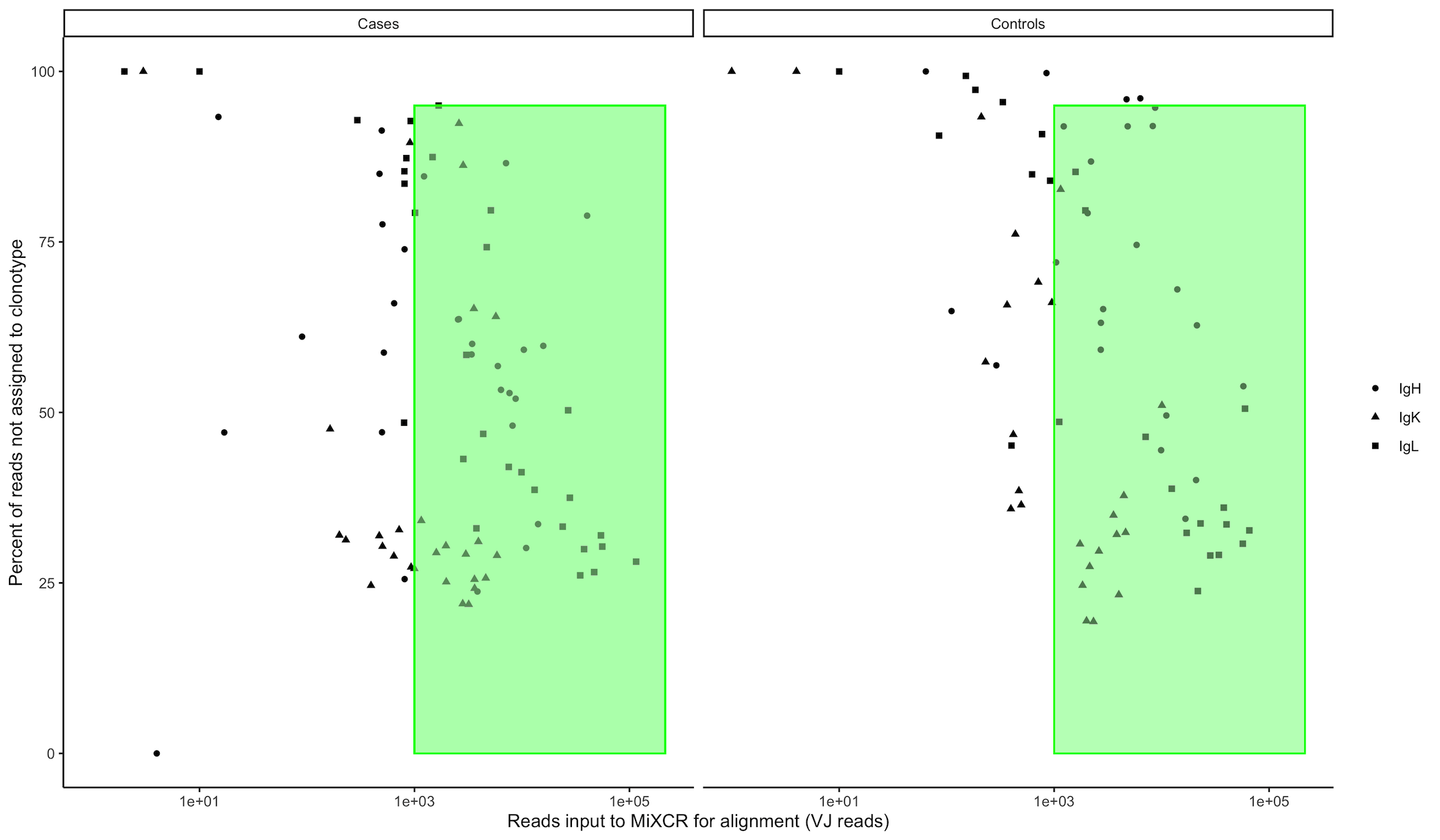

Supplement: S3 Fig — Percentage of VJ reads not assigned to a clonotype by the count (log scale) of VJ reads for each chain. Green box represents included chains with greater than 1000 reads and a maximum of 95% of reads not assigned to a clonotype by MiXCR. (TIFF) [file pone.0270710.s003.tiff]

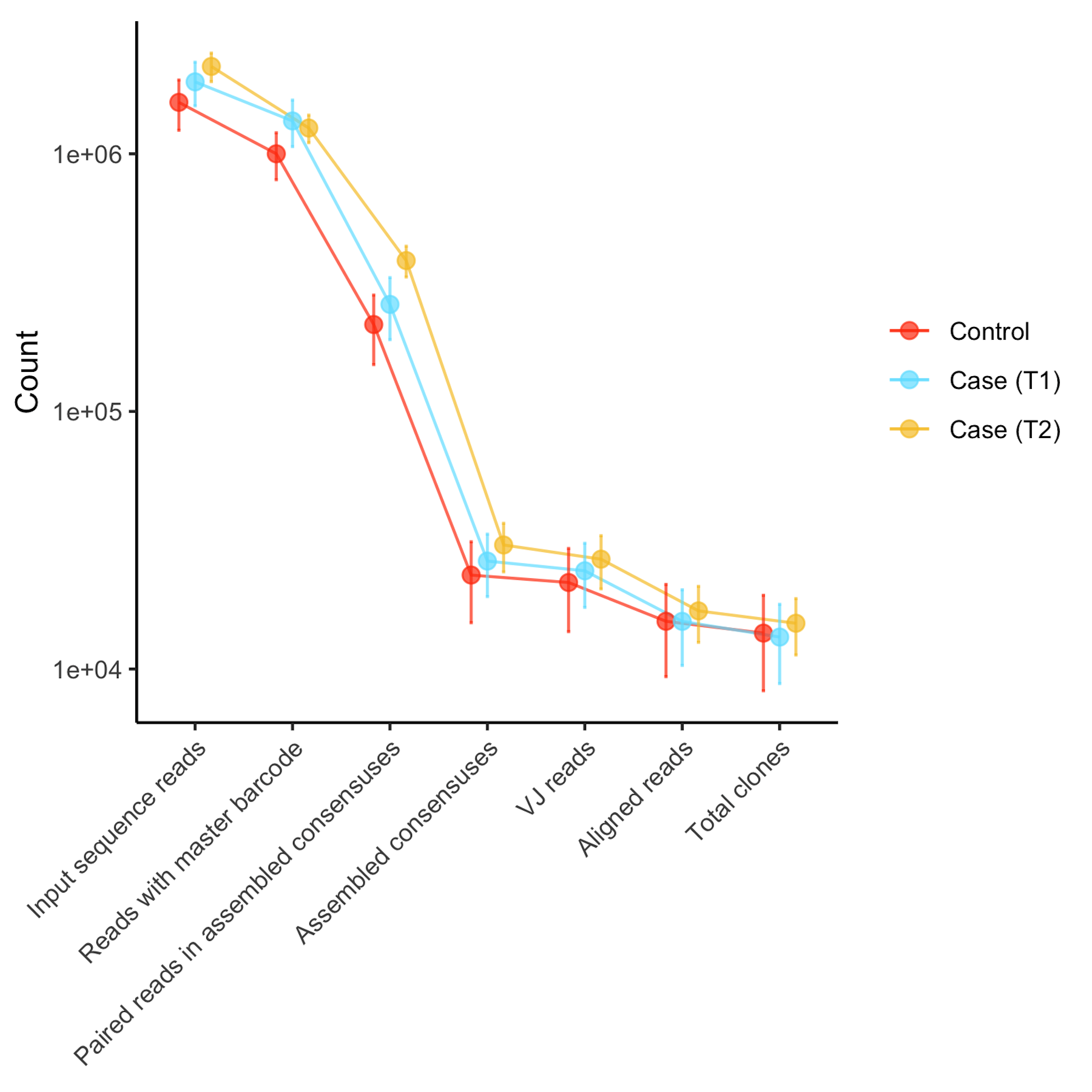

Supplement: S4 Fig — Decrease (log scale) in sequencing reads starting with raw input to reads containing master barcode (e.g. IGM, IGG, IGA, IGE, IGK, IGL), number of reads in assembled consensus sequences, total consensus sequences, VJ reads (potential clones), VJ reads successfully aligned again IMGT database, and total number of clones. Note the decrease from “Reads in assembled consensuses” to “Assembled consensuses” is not a filtering but a collapse. (TIFF) [file pone.0270710.s004.tiff]

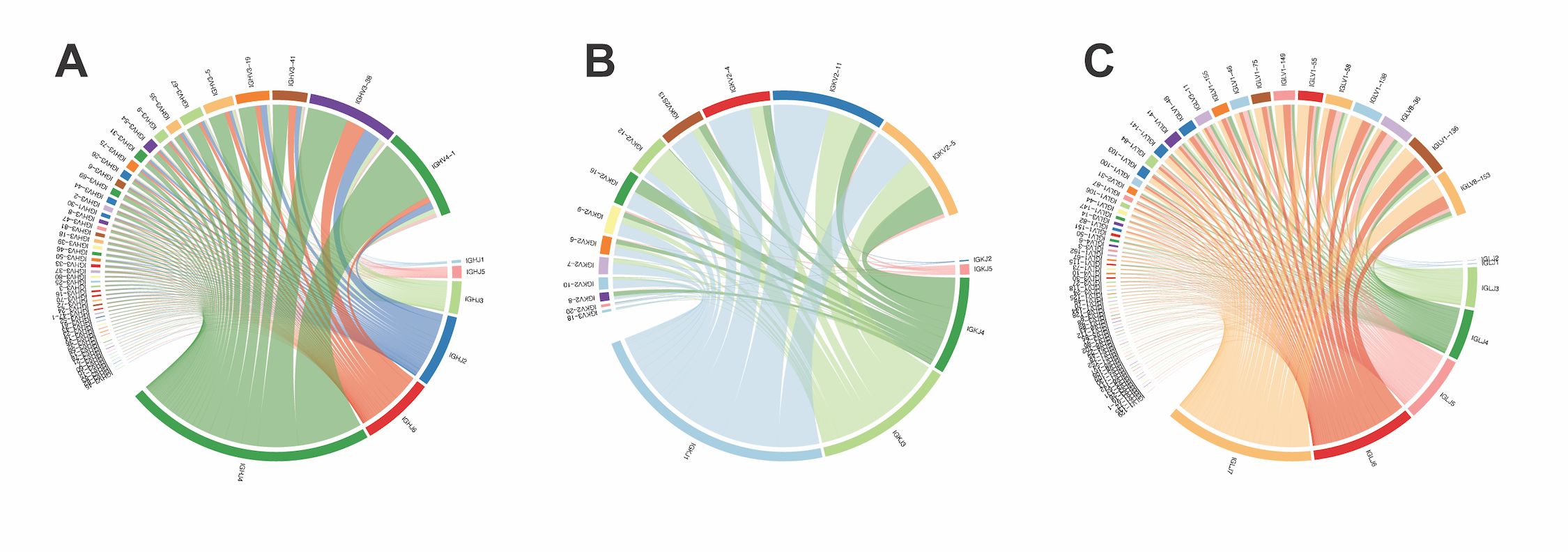

Supplement: S5 Fig — Around the outer edge of each plot are the V and J genes from each of the (A) IGH, (B) IGK, and (C) IGL loci. The thickness of the band joining any pair of V and J genes on the plot represents the unweighted proportion of reads that used that combination of V and J genes. (TIFF) [file pone.0270710.s005.tiff]

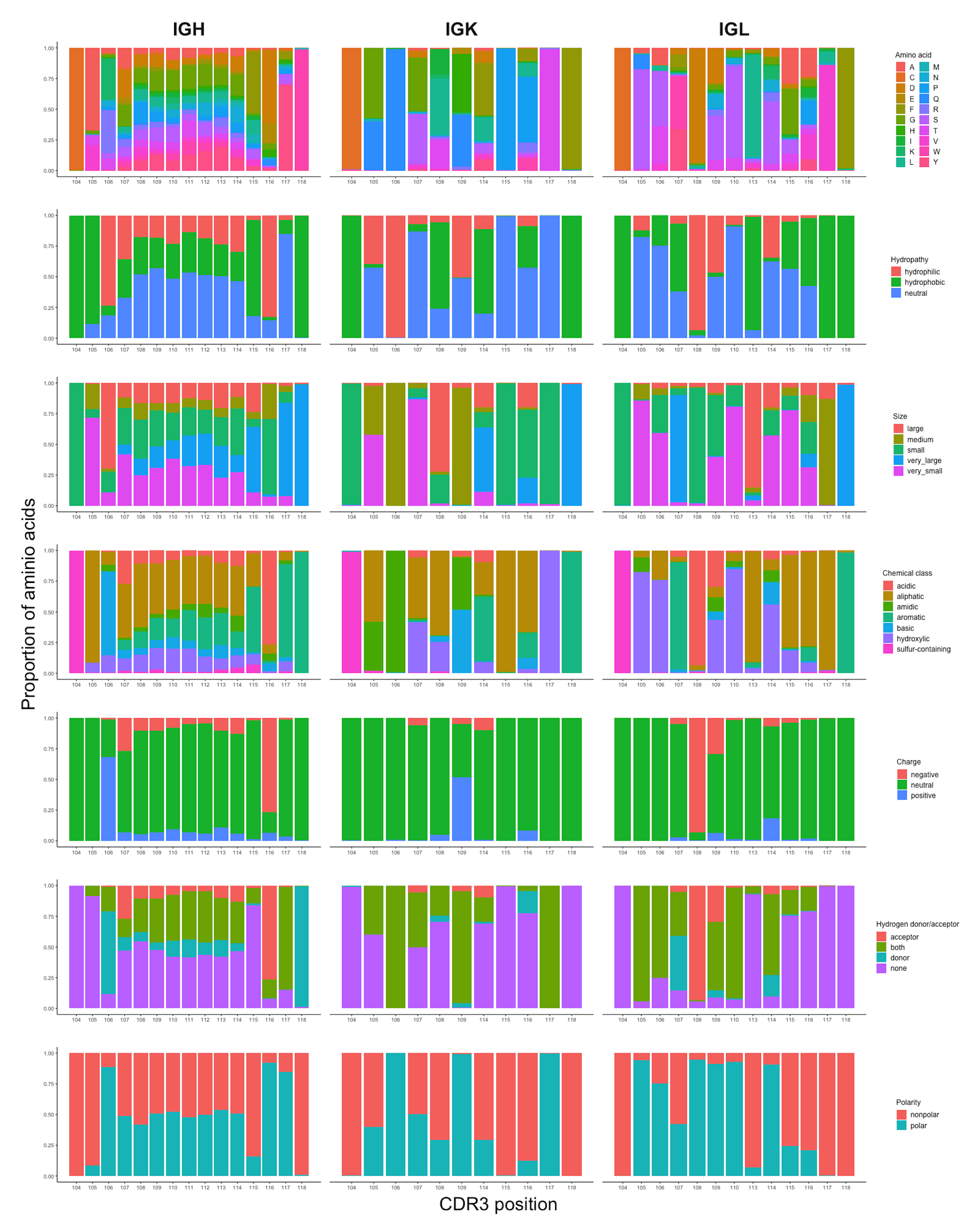

Supplement: S6 Fig — Reported properties at each CDR3 position, including hydropathy, size, chemical class, charge, hydrogen donor or acceptor class, and polarity were calculated based upon the proportion of amino acids at each position averaged across included samples using standard biochemical properties [34]. Only CDR3s of the most common length for (IGH = 13, IGK = 9, and IGL = 11) were considered. (TIFF) [file pone.0270710.s006.tiff]

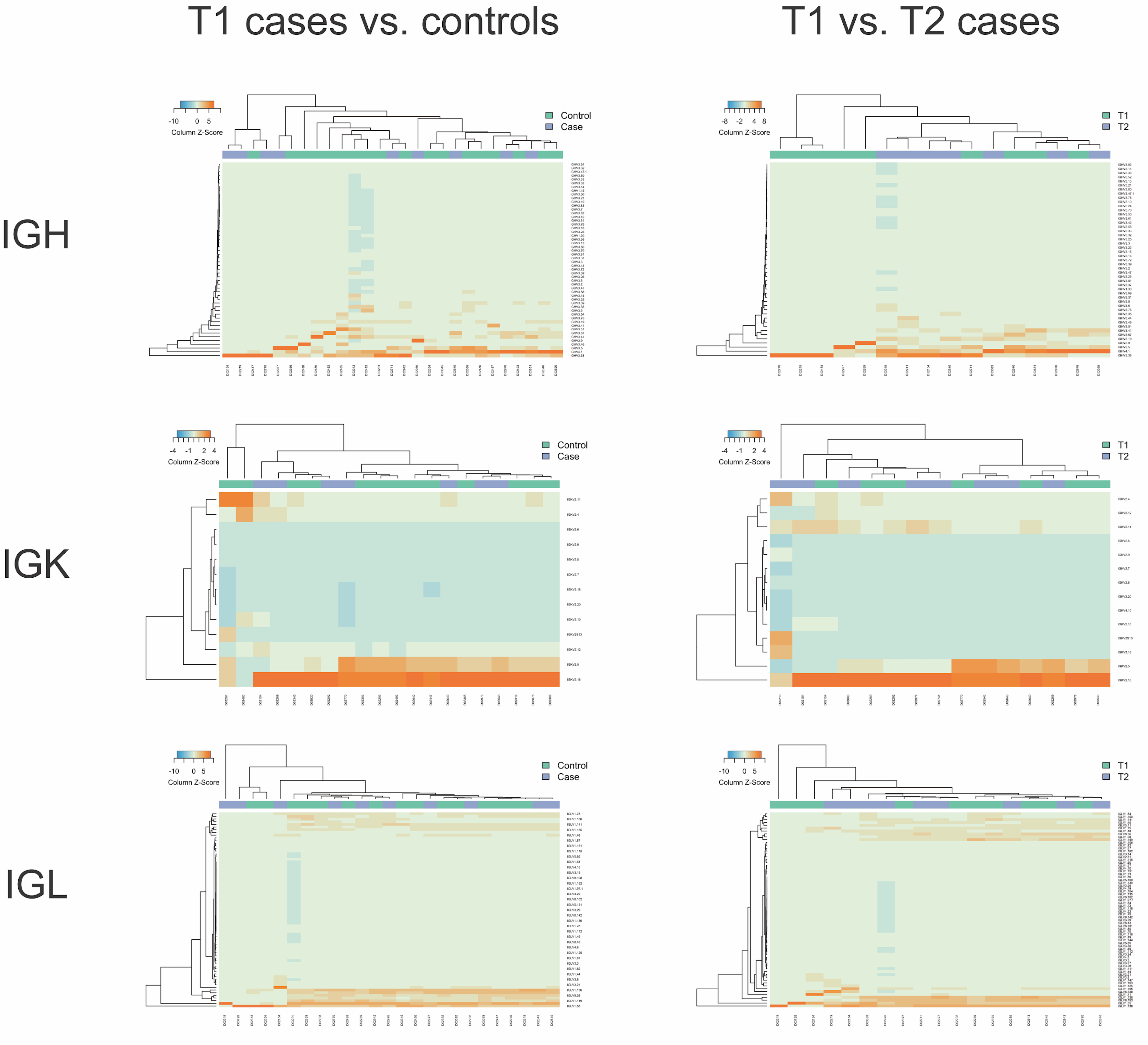

Supplement: S7 Fig — For each chain, V gene usage profiles are clustered using VDJtools’ “CalcSegmentUsage” method to compare both T1 cases and controls (left), and T1 and T2 cases (right) [28]. (TIF) [file pone.0270710.s007.tif]

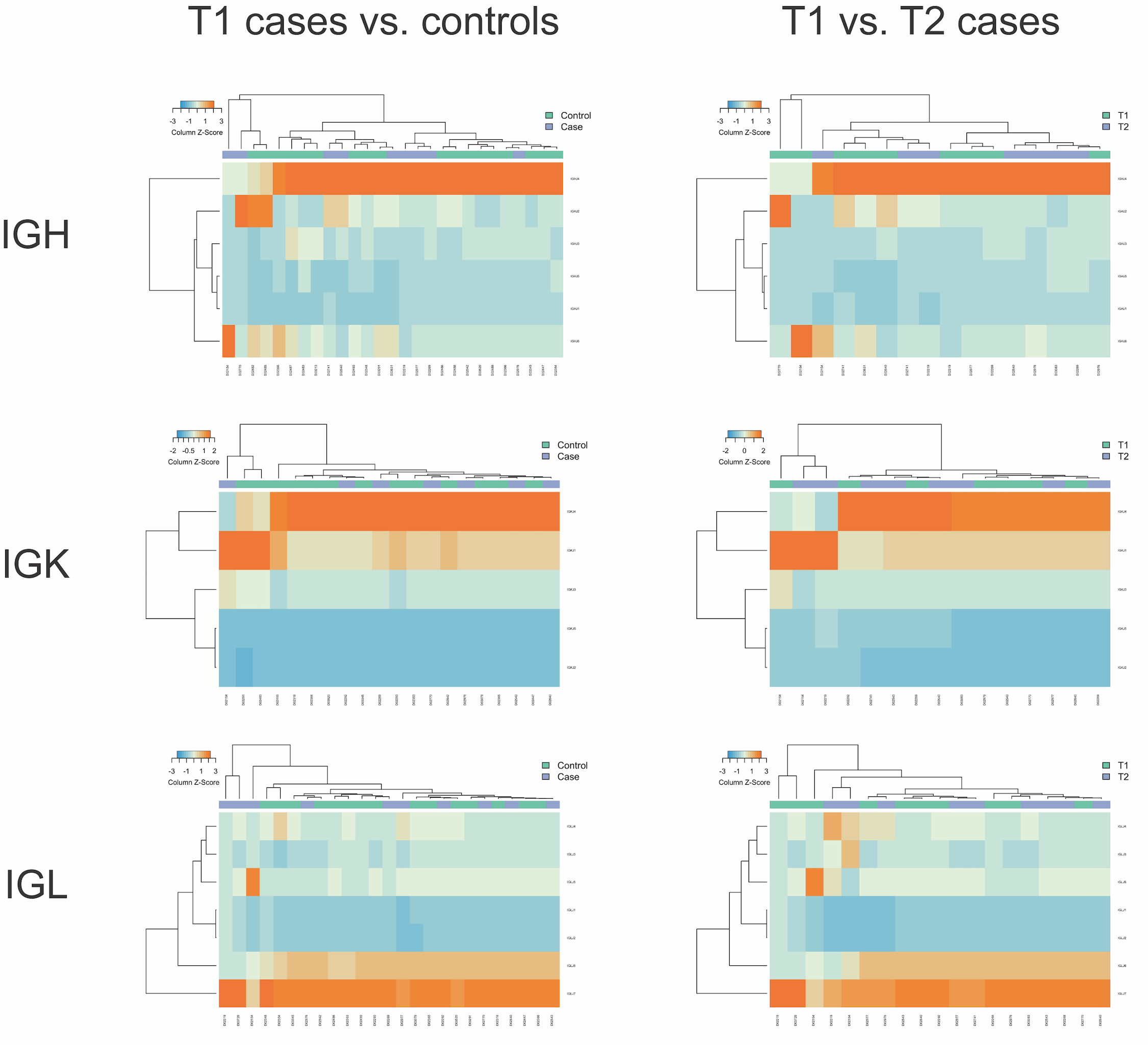

Supplement: S8 Fig — For each chain, J gene usage profiles are clustered using VDJtools’ “CalcSegmentUsage” method to compare both T1 cases and controls (left), and T1 and T2 cases (right) [28]. (TIF) [file pone.0270710.s008.tif]

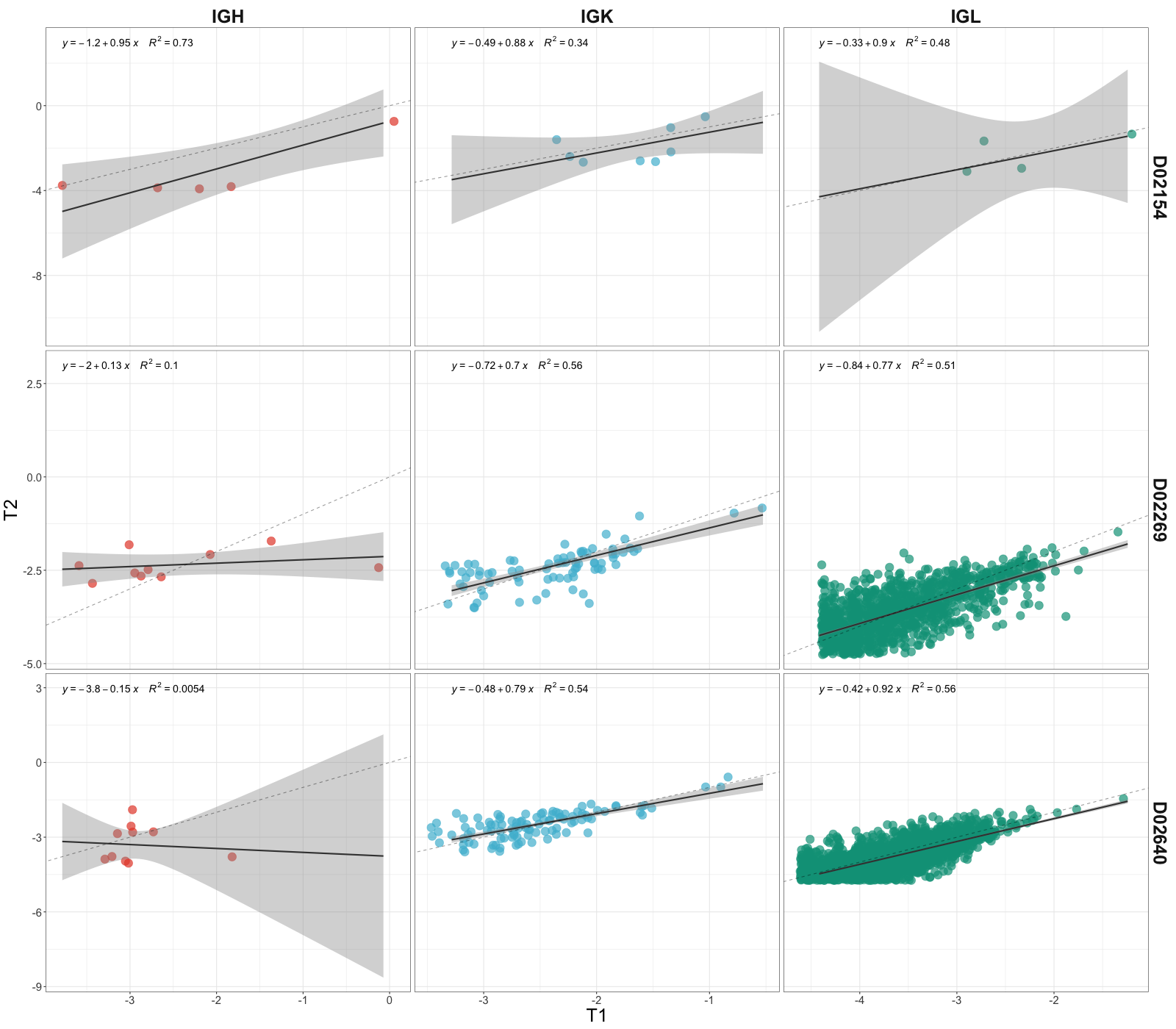

Supplement: S9 Fig — Clonotypes shared across time points are plotted for three cases (D02154, D02269, and D02640) where all three chains passed filtering criteria. The fitted regression equation and R2 are included. Jitter is applied to the data points. Dashed line (through the origin with slope 1) represents perfect concordance between T1 and T2 clonotypes (i.e. clonotypes observed at the same frequency in T1 and T2). Deviations from the dashed line indicate differences in clonotype frequency. VDJtools’ “OverlapPair” was used to identify shared clonotypes based on CDR3 amino acid sequence and V and J gene [28]. (TIFF) [file pone.0270710.s009.tiff]

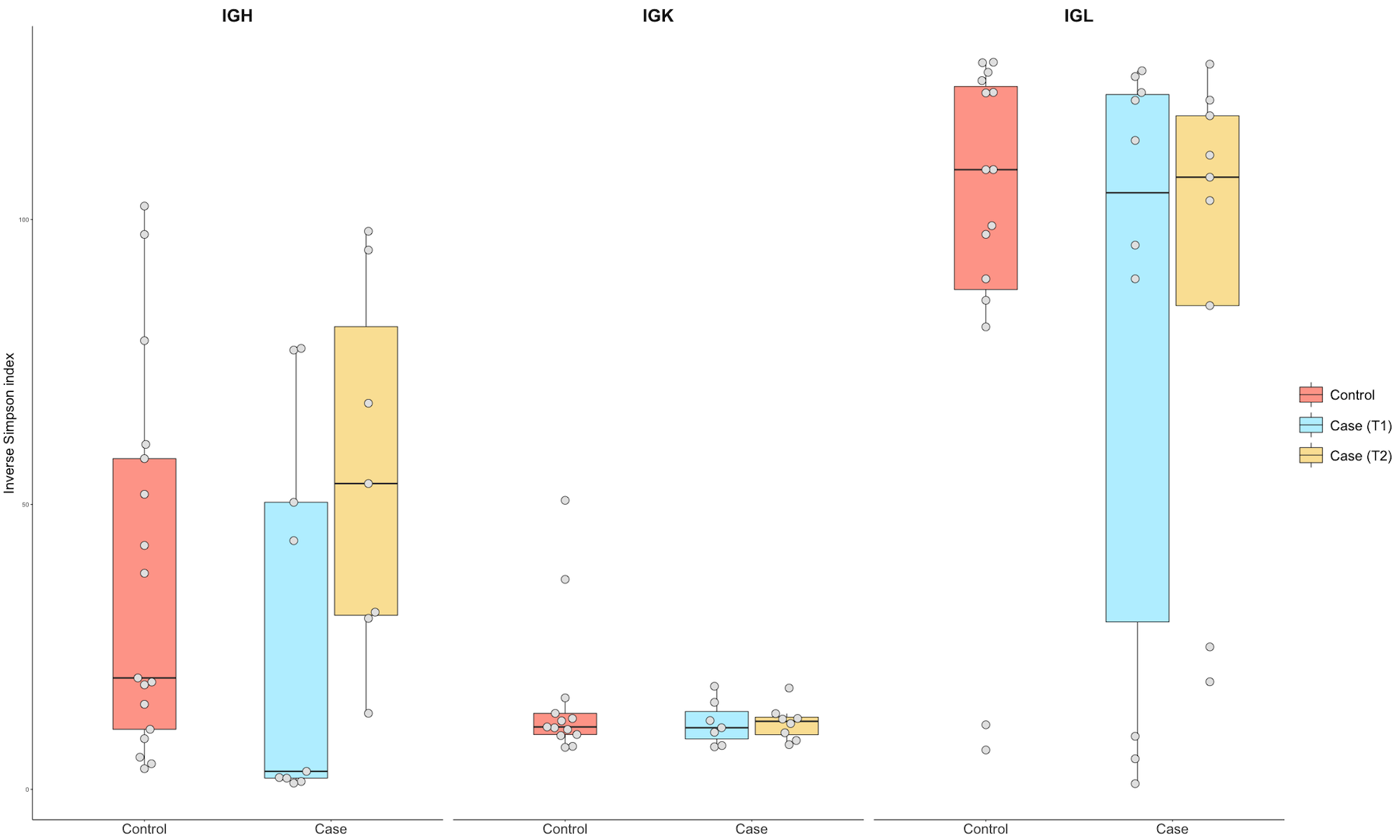

Supplement: S10 Fig — Inverse Simpson index as calculated by VDJtools “CalcDiversityStats” method for controls, and T1 and T2 cases across all chains [28]. Samples with clone size less than 100 were excluded in order to increase the accuracy of the resampling estimates. Refer to Table 1 for samples with paired comparisons between T1 and T2 (6 IGH, 4 IGK, and 7 IGL). (TIFF) [file pone.0270710.s010.tiff]

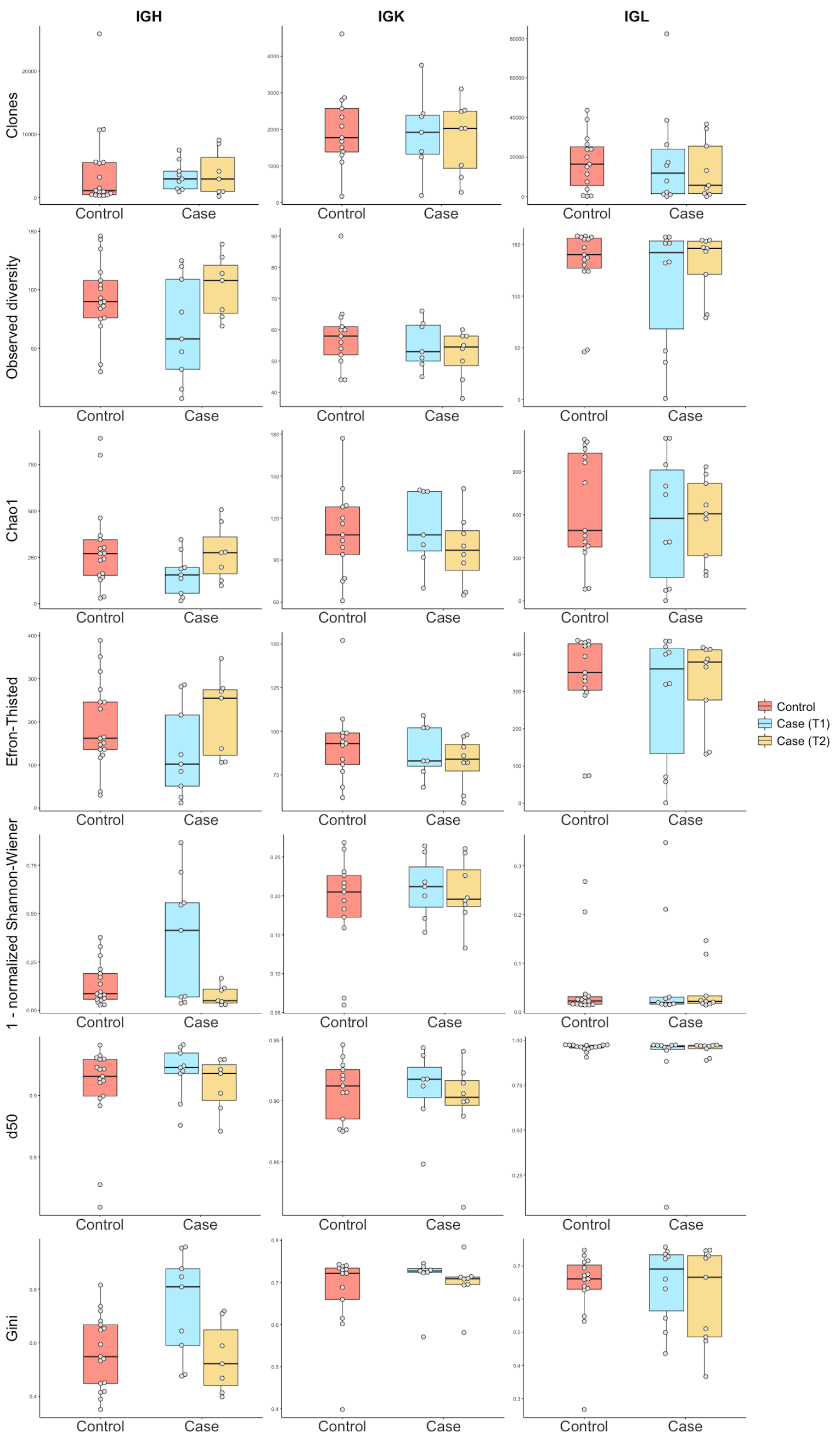

Supplement: S11 Fig — Control, T1 and T2 case diversity estimates of observed diversity, Chao1, Efron-Thisted, normalized Shannon-Wiener, and d50 by VDJtools’ “CalcDiversityStats” method [28]. Number of clones and the Gini index (calculated with the R package ineq [46]) are also included. D02219 had only two reported IGL clonotypes at T1 thus precluding the ability to calculate the normalized Shannon-Wiener index by VDJtools. (TIFF) [file pone.0270710.s011.tiff]

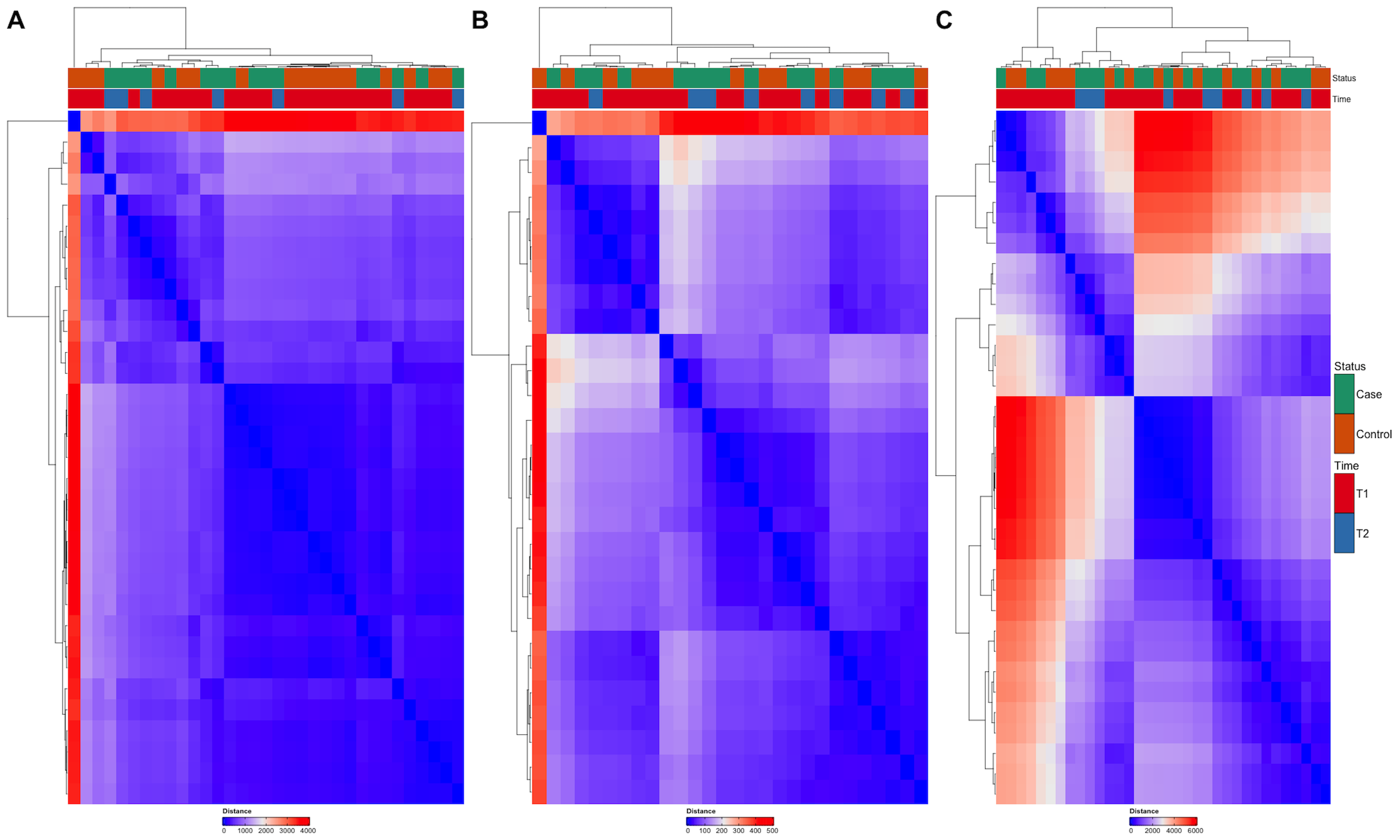

Supplement: S12 Fig — Euclidean distances were calculated pairwise between diversity profiles, followed by complete-linkage clustering, and visualization with ComplexHeatmap [44]. Case status and time point are included as annotations by color for (A) IGH, (B) IGK, and (C) IGL. (TIFF) [file pone.0270710.s012.tiff]

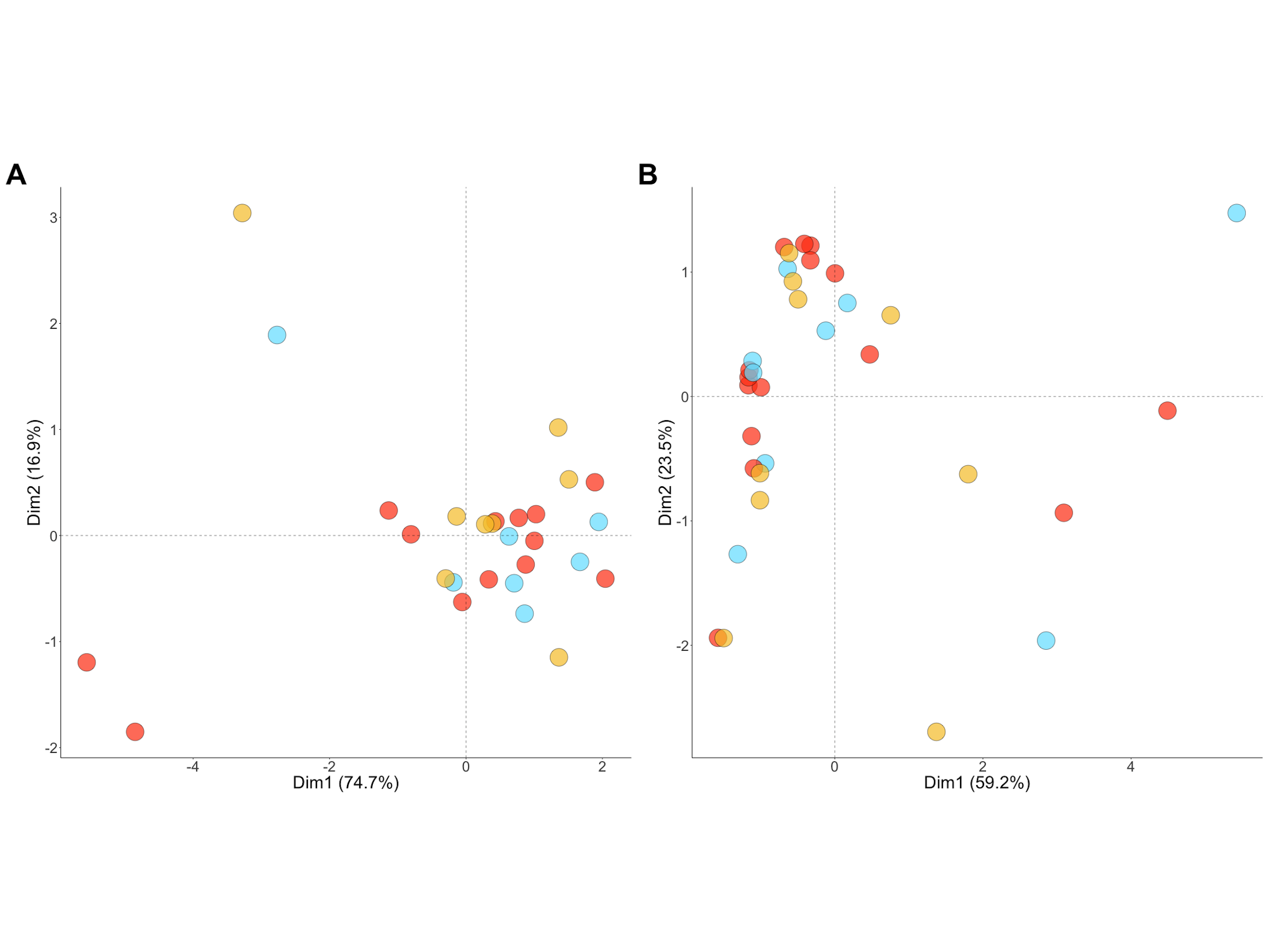

Supplement: S13 Fig — (A) IGK. (B) IGL. The normalized Shannon-Wiener index could not be calculated by VDJtools for D02219 at T1 and thus the single time point was removed. (TIFF) [file pone.0270710.s013.tiff]

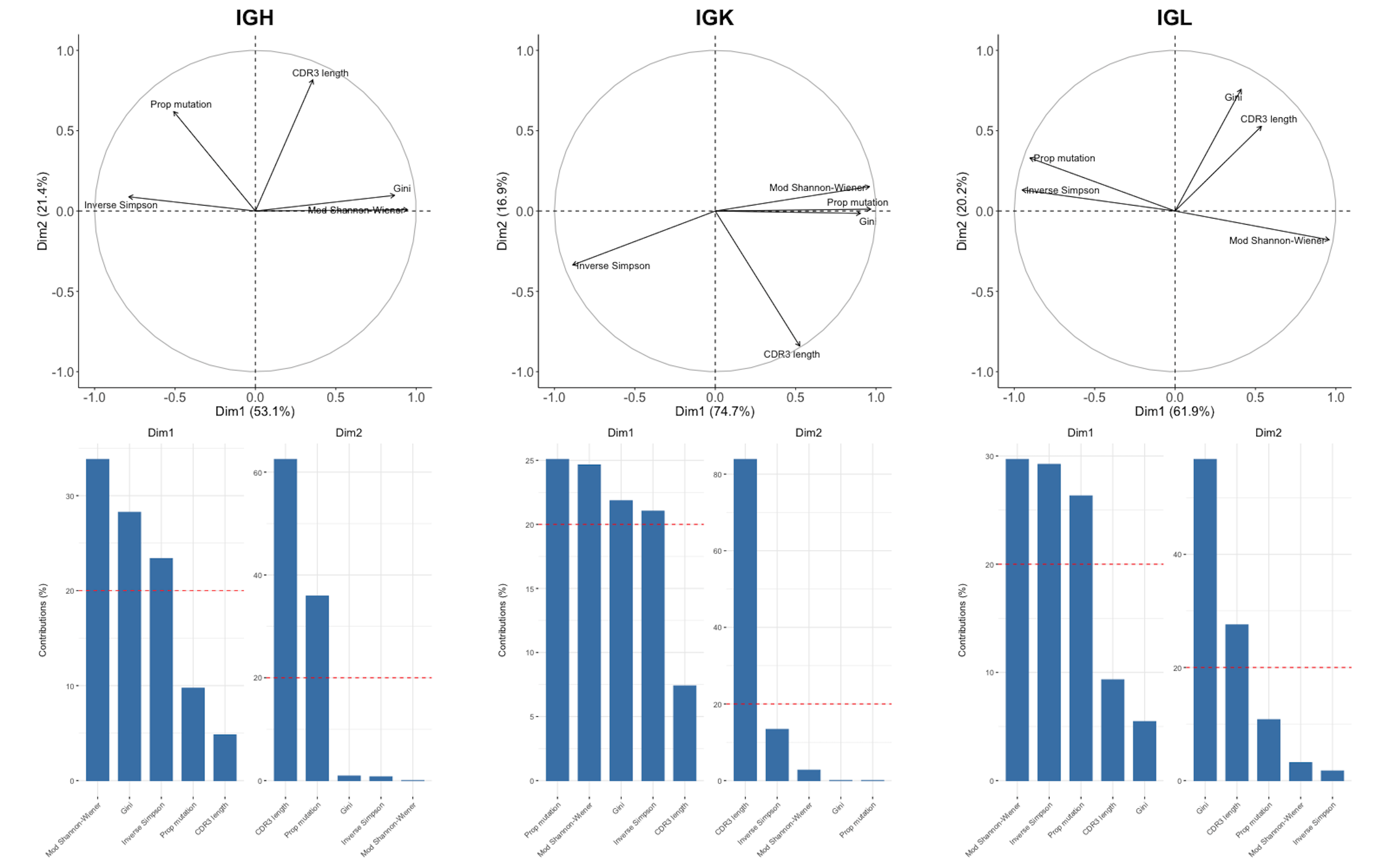

Supplement: S14 Fig — Percent contribution of each variable along the first and second principal components. (TIFF) [file pone.0270710.s014.tiff]
